# Nanomolar vanillin, an e-cigarette flavorant, appears to disrupt pluripotency and promote endodermal differentiation in human embryonic stem cells via TRPV4 activation

**DOI:** 10.1093/humrep/deag126

**Published:** 2026-08-12

**Authors:** Shabnam Etemadi, Prue Talbot

**Affiliations:** Department of Bioengineering, University of California, Riverside, CA, USA; Department of Molecular, Cell and Systems Biology, University of California, Riverside, CA, USA

**Keywords:** human embryonic stem cells, vanillin, epiblast, early embryonic development, TRPV4 channel, gastrulation, cell death, proliferation, differentiation, electronic cigarettes

## Abstract

**STUDY QUESTION:**

Does vanillin, at concentrations relevant to maternal exposures during vaping, disrupt gastrulation-related processes in human embryonic stem cells (hESCs) by activating TRPV4 channels?

**SUMMARY ANSWER:**

Activation of TRPV4 channels by nanomolar concentrations of vanillin promoted an exit from pluripotency and a shift toward endodermal gene expression, while micromolar concentrations induced developmental toxicity, identifying a concentration-dependent mechanism by which vanillin, a flavoring agent in electronic cigarette (EC) aerosols, may pose risks to embryonic development during pregnancy.

**WHAT IS KNOWN ALREADY:**

The use of ECs during pregnancy is increasing, potentially driven by perceptions of reduced harm and the appeal of flavoring agents, such as vanillin. However, vanillin activates transient receptor potential (TRP) channels, raising concerns about its impact on early embryonic development.

**STUDY DESIGN, SIZE, DURATION:**

hESCs, which model the epiblast stage of development, were treated *in vitro* for various times with vanillin, a TRPV4 antagonist, a TRPV4 function-blocking antibody, or vanillin combined with either the antagonist or antibody. All experiments were done three times with different passages of stem cells.

**PARTICIPANTS/MATERIALS, SETTING, METHODS:**

After variable periods of exposure, the hESC colonies were evaluated for activation of TRPV4 channels and calcium influx, colony growth, colony detachment, increased cell death, mitochondrial dysfunction, colony morphology, gap formation, downregulation of EpCAM, loss of pluripotency, and initiation of differentiation.

**MAIN RESULTS AND THE ROLE OF CHANCE:**

We demonstrate that vanillin, at concentrations relevant to maternal EC exposure, significantly (*P* < 0.05) disrupted gastrulation-related processes in hESCs. hESCs, which model the epiblast during Weeks 2–3 of human development, exhibited TRPV4 channel expression and showed a significant increase in intracellular calcium upon nanomolar-to-micromolar vanillin exposure, as detected by the Fluo-8 calcium dye. Time-lapse imaging and quantitative analysis indicated that micromolar vanillin impaired colony expansion, induced colony detachment, and increased cell death over 72 h, while concurrently reducing mitochondrial reductase activity in the MTT assay. At nanomolar concentrations, vanillin promoted TRPV4 activation, an exit from pluripotency, downregulation of EpCAM cell adhesion protein, intercellular gap formation, and a shift toward endodermal gene expression. All effects were blocked by a TRPV4 antagonist (HC067047), identifying activation of this channel as a critical mediator of vanillin-induced toxicity.

**LARGE SCALE DATA:**

N/A.

**LIMITATIONS, REASONS FOR CAUTION:**

Our data are derived from *in vitro* experiments, as direct investigation of early human development is ethically and practically limited. Although our findings do not establish embryo lethality or malformation *in vivo*, the observed loss of pluripotency and SOX17 upregulation, consistent with endodermal induction, at nanomolar concentrations of vanillin are signs of early cellular perturbations that could compromise human development.

**WIDER IMPLICATIONS OF THE FINDINGS:**

Nanomolar vanillin concentrations, which our exposure model estimates reach the embryo during early pregnancy in EC users, were sufficient to activate TRPV4 channels in hESCs and alter germ layer specification. Micromolar concentrations produced cytotoxic effects and increased cell death. These findings indicate that vanillin exposure at levels attainable during EC use disrupts intracellular Ca^2+^ homeostasis and compromises core processes of early human development. Maternal EC use during pregnancy therefore represents a significant and previously under-appreciated risk to early embryogenesis. Clinicians should counsel patients who are pregnant or planning pregnancy about this potential harm, and regulatory agencies should consider mandatory ingredient disclosure on EC packaging.

**FUNDING:**

This research was supported by grant number T32IR4848 from the Tobacco-Related Disease Research Program (TRDRP), by grant number EDUC4-12752 from the California Institute of Regenerative Medicine (CIRM), and by UCR Yvonne Danielson Endowed Graduate and Dissertation Completion Fellowship Awards. The content is solely the responsibility of the authors and does not necessarily represent the official view of the TRDRP, CIRM, or UCR.

**DISCLOSURES:**

The authors have no conflicts of interest to declare.

## Graphical Abstract

**Graphical Abstract:**
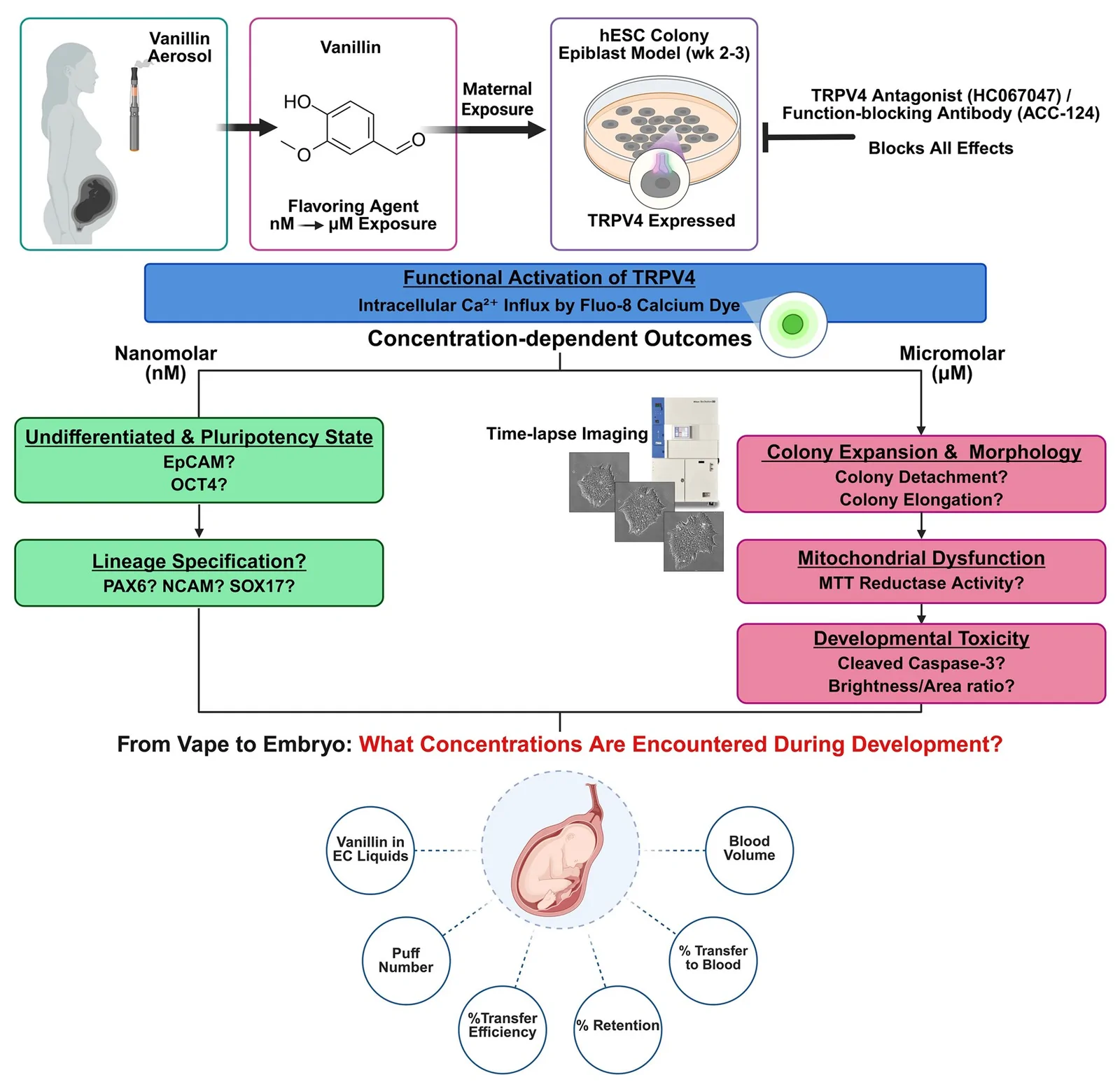
**Human embryonic stem cells (hESCs) were used as a model of epiblast development to investigate the effects of varying concentrations of vanillin on transient receptor potential vanilloid 4 (TRPV4) channels and indicators of cell growth and differentiation.** EC, electronic cigarette. Created in BioRender. Etemadi, S. (2026) https://BioRender.com/hafq41e.

## Introduction

Maternal smoking is a well-established risk factor for adverse pregnancy outcomes, prompting some reproductive-aged women to switch to electronic cigarettes (ECs), which are perceived as safer alternatives (Wagner *et al.*, 2017; Tarasi *et al.*, 2022; Lutman-White *et al.*, 2024). However, epidemiological data from large-scale studies (e.g. PRAMS, PATH) have found associations between EC use during pregnancy and increased risks characterized by low birthweight, pre-term birth, small for gestational age, fetal growth restriction and neurological dysfunction (Froggatt *et al.*, 2020; Cohn *et al.*, 2023; Bailey *et al.*, 2024; Bell *et al.*, 2025; Deprato *et al.*, 2025). Despite these associations, little is known about how specific EC constituents, such as flavorants which are often used at very high concentrations (Behar *et al.*, 2018; Omaiye *et al.*, 2019a), affect early human development at the cellular and molecular levels.

Vanillin (4-hydroxy-3-methoxybenzaldehyde) is a widely used flavorant in EC refill fluids (Tierney *et al.*, 2016; Hua *et al.*, 2019; Omaiye *et al.*, 2020, 2022b; Khachatoorian *et al.*, 2022), second- and third-generation vaping devices, such as vape pens and box mods (Behar *et al.*, 2018), fourth-generation pod-based systems (e.g. JUUL) (Omaiye *et al.*, 2022a,b), and disposable products (e.g. PUFF Bar) (Omaiye *et al.*, 2022a,b). It is found in over half of the tested e-liquids and can reach concentrations that sometimes exceed 30 mg/ml (Tierney *et al.*, 2016; Behar *et al.*, 2018), far higher than levels typically used in foods or cosmetics (≤ 0.01 mg/ml; Joint FAO/WHO Expert Committee on Food Additives, 2001; Omaiye *et al.*, 2022a,b). Human EC inhalation studies show high transfer efficiency and pulmonary retention of aerosol constituents, including vanillin (Behar *et al.*, 2018; Khachatoorian *et al.*, 2022; Hua *et al.*, 2023).

Given the high concentrations of vanillin detected in EC aerosol constituents, we hypothesized that uterine exposure in women who vape could reach embryotoxic thresholds sufficient to impair early embryonic development. A candidate mechanism through which inhaled vanillin may exert developmental effects is activation of transient receptor potential vanilloid 4 (TRPV4), a calcium-permeable ion channel with broad expression in epithelial and embryonic tissues (Lübbert *et al.*, 2013; Premkumar, 2014; Oka *et al.*, 2022). While direct activation of TRPV4 by vanillin has not been reported, TRPV4 is a biologically relevant candidate given its expression in embryonic and epithelial tissues where it regulates lung morphogenesis, epithelial differentiation, and fetal inflammatory responses (Nayak *et al.*, 2015; Morgan *et al.*, 2018). Moreover, in our prior screen of an RNA sequencing library, human embryonic stem cells (hESCs) had significant expression of TRPV4 (Etemadi and Talbot, 2025). During gastrulation, when germ layers are specified, activation of TRPV4 by vanillin may compromise embryo viability, pluripotency, and germ cell specification.

The purpose of this study was to examine the susceptibility of hESCs to vanillin at concentrations likely to reach the embryo in pregnant women who vape. hESCs are a well-established model for the epiblast (Nichols and Smith, 2009, 2011): a post-implantation tissue that undergoes gastrulation during Weeks 3–4 of human development (Oh *et al.*, 2023). We first investigated the effects of micromolar versus nanomolar vanillin concentrations on TRPV4 channel activation and its impact on downstream processes critical to gastrulation. We then used a quantitative embryo exposure model to show overlap between vanillin concentrations that produce adverse effects in hESCs *in vitro* and concentrations likely to reach an embryo in pregnant women who use ECs containing vanillin.

## Materials and methods

### Chemicals, reagents, antibodies, and supplies

The supplies and chemicals used in this project, including company names, catalogue numbers, and dilutions are presented in Supplementary Table S1.

### hESC cell culture

H9 and H1 hESCs were cultured in mTeSR™ Plus medium on wells coated with hESC-qualified Matrigel, as previously described (Lin and Talbot, 2011; Behar *et al.*, 2012). Cells were maintained at 37 °C in humidified incubators with 5% CO_2_ and monitored daily to ensure normal morphology. For subculturing, hESCs at 70–80% confluency were first rinsed with calcium- and magnesium-free Dulbecco’s phosphate-buffered saline (DPBS), then incubated with ReLeSR for 5 min to dissociate the colonies, followed by a 1-min Accutase® incubation for enzymatic cell detachment. Cells were pelleted by centrifugation, counted using a BioMate 3S Spectrophotometer, and seeded at densities of 2 × 10^4^ cells/well in 96-well plates, 5 × 10^4^ cells/chamber in 8-well chamber slides, and 1 × 10^5^ cells/well in 12-well plates. The authenticity of hESC lines was verified by short tandem repeat profiling (WiCell, SOP-CH-305), and routine testing confirmed that cultures were free of Mycoplasma contamination. To investigate the functional role of TRPV4 channels, both a pharmacological antagonist (HC067047) and a TRPV4 function-blocking antibody (ACC-124) were used (Supplementary materials and methods). Cells were pre-incubated with either the antagonist or antibody for 30 min prior to vanillin treatment and remained in the culture medium during the treatment.

### Immunocytochemistry labeling and analysis

To evaluate protein expression, H9 and H1 hESCs were plated in 8-well ibidi chamber slides. Upon reaching ∼80% confluency or following 3–6 days of vanillin treatment, cells were fixed in 4% paraformaldehyde for 30 min at room temperature followed by an extensive wash with DPBS. Immunocytochemistry labeling was then performed as detailed in Supplementary materials and methods. The presence and distribution of TRPV4 and TRPV1 channels were studied using rabbit polyclonal antibodies to human TRPV4 and TRPV1, respectively.

### Calcium influx assay

Calcium influx induced by vanillin (0.45–100 µM) and vanillic acid (100 µM) was quantified using the Fluo-8 No Wash Calcium Assay Kit, following the manufacturer’s protocol. H9 and H1 hESCs were grown to 70–80% confluency in 96-well clear-bottom black plates. They were then incubated with 1 µM Fluo-8 in HHBS buffer for 20 min at 37 °C. Baseline fluorescence was recorded for 20 s, after which measurements were taken for 4 min following the addition of vanillin at 37 °C using a Synergy HTX Microplate Reader. Changes in intracellular calcium levels were analyzed using MATLAB R2024b and plotted using GraphPad Prism.

### Assessment of mitochondrial reductase activity by MTT assay

The MTT assay was used to evaluate the effect of vanillin on mitochondrial reductases in H9 hESCs. Cells in 96-well clear plates were seeded and allowed to attach 48 h before treatment with vanillin (45 µM–45 mM) for 24 h. The MTT assay procedure followed the protocol detailed in Supplementary materials and methods.

### Time-lapse imaging and colony analysis

Time-lapse imaging was performed to evaluate colony growth (normalized area), colony morphology (major/minor axis ratio, gap formation), and colony death (detachment, brightness/total area) using a Nikon BioStation CT equipped with a 10× phase contrast objective and automatic Z-focus as previously described (Talbot *et al.*, 2014) and detailed in Supplementary materials and methods.

### Cell death assay

To evaluate the effect of 45–450 µM vanillin on cell death, H9 and H1 hESCs were immunolabeled with an antibody to activated caspase-3, a cysteine-aspartic acid protease involved in the execution phase of apoptosis (Shi, 2002). The percentage of positively stained cells was quantified using the Cell Counter plugin in ImageJ, as described previously (Etemadi *et al.*, 2025).

To confirm cell death, a second validated assay was used in which the ratio of the colony brightness to total colony area was measured in phase contrast images (Zahedi *et al.*, 2016; Lin *et al.*, 2021; Etemadi *et al.*, 2025). The brightness/total area protocol is included in Supplementary materials and methods.

### Cell proliferation assay

Following treatment with 45–450 µM vanillin, proliferation of H9 and H1 hESCs was evaluated by scoring the mitotic index. Mitotic stages (metaphase and anaphase) were identified by their distinct nuclear morphology following DAPI staining, as previously described (Etemadi *et al.*, 2025). To exclude apoptotic cells from the analysis, samples were co-immunostained for activated (cleaved) caspase-3 prior to quantification. Mitotic cells were then counted using the Cell Counter plugin in ImageJ.

### Cell-to-cell gap analysis

Intercellular gap analysis was performed using an antibody specific to epithelial cell adhesion molecule (EpCAM): a single-pass membrane glycoprotein involved in cell–cell adhesion. H9 hESCs were plated in 8-well chamber slides and allowed to grow for 48 h before treatment with vanillin at concentrations of 0.45–45 µM for 72 h. Following exposure, cells were immunolabeled with a mouse monoclonal antibody against human EpCAM (CD326). EpCAM staining was quantified in colonies of ∼100 cells. Using the CL-Quant Procedures menu, masks were generated for each colony to measure dot area, total area, and mean fluorescence intensity. All masks were manually reviewed and edited to exclude background fluorescence.

### Pluripotency and differentiation analysis

The impact of vanillin on stem cell pluripotency and differentiation was investigated by immunostaining H9 hESCs for specific markers. hESCs were seeded in 150 µl of mTeSR™ Plus medium in 8-well chamber slides and allowed to attach for 48 h. Cells were then treated with 0.45 µM (450 nM) vanillin for 6 days at 37 °C. Following treatment, cells were fixed in 4% paraformaldehyde and immunostained for markers of pluripotency and germ layer differentiation.

Germ layer-specific cells (ectoderm, mesoderm, and endoderm) were generated as positive controls for comparison with the hESCs treated with nM vanillin for 6 days. The tri-lineage differentiation protocol is described in Supplementary materials and methods. Correct differentiation was validated using established markers for each germ layer (Supplementary Fig. S1): PAX6-labeled ectoderm, NCAM-labeled mesoderm and SOX17-labeled endoderm, confirming appropriate lineage specification. The pluripotency marker OCT4 was absent in all three germ layers, consistent with successful differentiation and loss of the pluripotent state. For analysis of pluripotency and germ layer markers, fluorescence intensity was quantified using ImageJ and expressed as integrated density (fluorescent signal mean divided by colony area). For each experimental group, measurements were obtained from three colonies/passage across three independent passages, and the resulting values were averaged. All image acquisition settings and analysis parameters were kept constant across experimental conditions.

### Exposure modeling and concentration relevance

Vanillin was tested *in vitro* at nanomolar and micromolar concentrations. To determine if these concentrations were likely to reach the embryo in a pregnant woman who vapes, we applied our quantitative embryo exposure model to our vanillin data. This model was previously used and validated with menthol and WS-23 (Etemadi and Talbot, 2025; Etemadi *et al.*, 2025). The model integrates vanillin levels reported in EC refill fluids (3–33 mg/ml) (Tierney *et al.*, 2016; Behar *et al.*, 2018), the efficiency with which vanillin transfers to the aerosol (∼70–98%) (Behar *et al.*, 2018; Khachatoorian *et al.*, 2022), pulmonary retention of vanillin in human EC users (∼95%) (Khachatoorian *et al.*, 2022; Hua *et al.*, 2023), the fraction of inhaled vanillin passing from the lungs into the maternal circulation (50%), and dilution of vanillin by maternal blood volume (∼2650 ml) (Aguree and Gernand, 2019). The model was applied using various puff numbers/hour to account for variations in user topography (Behar *et al.*, 2015; Hua *et al.*, 2023). We also ran the model with various concentrations of vanillin in e-liquids to reflect the range that has been reported in EC products (Tierney *et al.*, 2016; Omaiye *et al.*, 2022a). Vanillic acid, the primary metabolite of vanillin, was tested at concentrations comparable to those used for vanillin and within the range previously reported in human plasma (Farthing *et al.*, 1999).

### Data analysis and statistics

All experiments were performed using three different passages of hESCs to obtain biological variation, and for each passage, three technical replicates were done. Data are presented as mean ± SEM. Statistical analyses were performed in GraphPad Prism (v10.4.2). Normality and homogeneity of variance were assessed prior to analysis; non-normally distributed data were log-transformed. One-way ANOVA followed by Dunnett’s *post hoc* test (vs untreated control) or Tukey’s (pairwise) *post hoc* test was applied to Ca^2+^ influx, colony morphology and detachment, cell death and proliferation, mitochondrial activity (MTT), intercellular gap formation, and pluripotency and germ layer differentiation marker expression. Time-lapse colony area data were analyzed using a two-way ANOVA with Dunnett’s *post hoc* test. Statistical significance was set at *P* < 0.05.

## Results

### Micromolar vanillin induces calcium influx in hESCs by activating TRPV4 channels

TRPV4 channels were present in H9 hESCs and appeared as bright green puncta on the surfaces of immunolabeled cells (Fig. 1A and B). There was no labeling when the secondary antibody alone was used (Fig. 1C).

**Figure 1.**
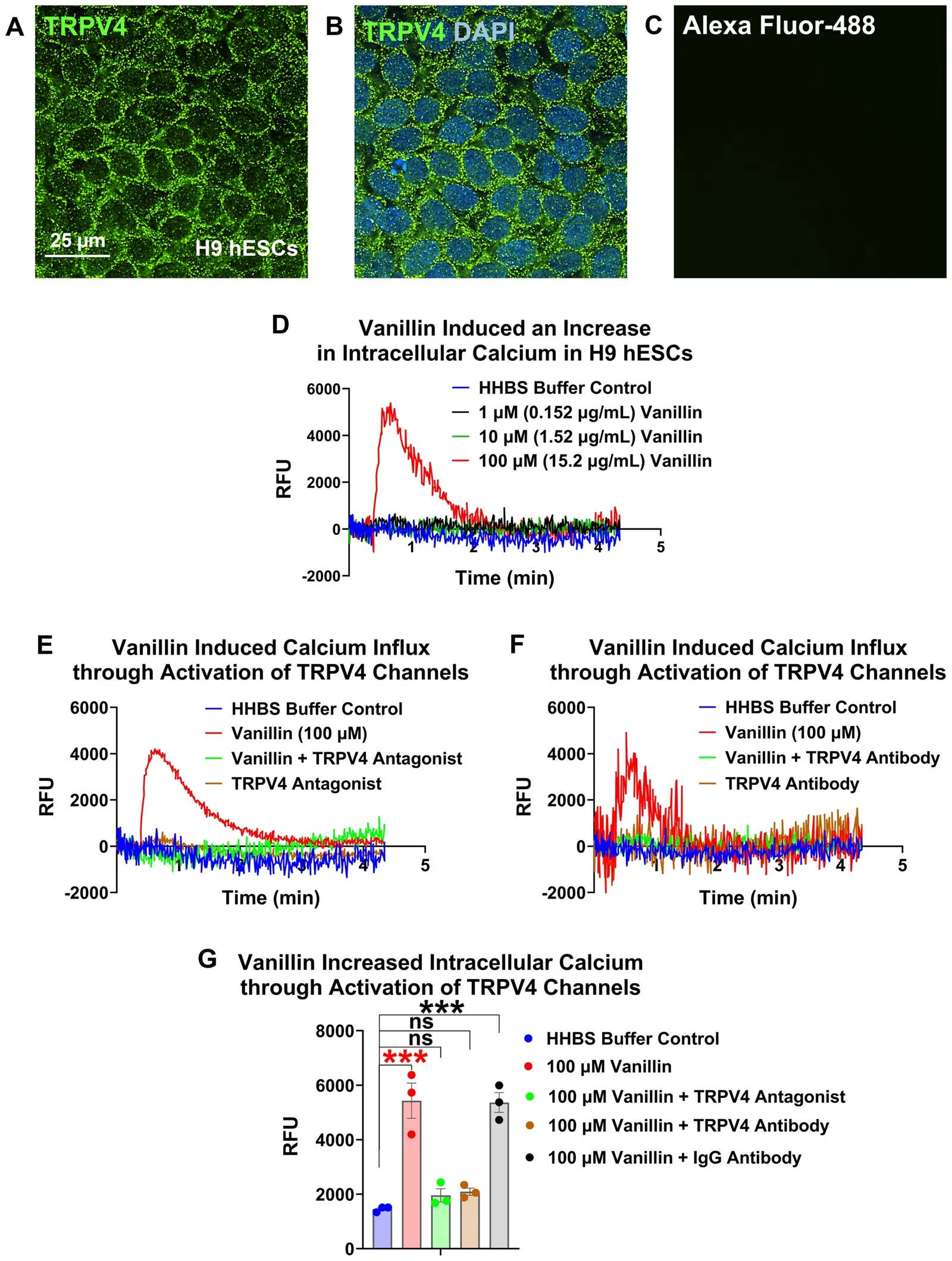
**Vanillin induces calcium influx in H9 hESCs by activation of TRPV4 channels.** (**A, B**) Immunofluorescence micrographs of hESCs labeled with a human TRPV4 antibody (green) counterstained with DAPI (blue) to visualize nuclei. (**C**) Negative control micrograph showing no fluorescence in cells labeled with secondary antibody only. Scale bar = 25 µm. Immunolabeling was performed in three independent experiments, each done with a different passage of cells. (**D**) Representative graph showing increased intracellular calcium levels in hESCs following exposure to vanillin. (**E**) Calcium influx induced by 100 µM vanillin was blocked by the TRPV4 antagonist HC067047 (0.1 nM), which itself did not affect intracellular calcium levels. (**F**) The TRPV4 function-blocking antibody ACC-124 (1 µM), but not the control IgG antibody, significantly reduced calcium influx induced by 100 µM vanillin. RFU (relative fluorescence units) for each graph was baseline subtracted. (**G**) Quantification of peak calcium responses for HHBS buffer control, vanillin, and vanillin plus TRPV4 antagonist or antibody. Bars represent the means±SEM for three independent experiments, each done with a different passage of cells. Statistical analysis was performed using one-way ANOVA followed by Dunnett’s *post hoc* test with comparisons to the control. ns = non-significant, ***P < 0.001.

We first tested vanillin at micromolar concentrations, previously shown to be bioactive in numerous toxicological studies (Stefaniak *et al.*, 2021). Exposure to 100 µM vanillin induced an immediate and significant increase in intracellular calcium [Ca^2+^]_i_, reaching a peak of ∼5000 RFUs within 40 s and returning to baseline within 4 min (Fig. 1D). Pre-treatment with the TRPV4 antagonist (HC067047) (Fig. 1E) or with the function-blocking antibody (Fig. 1F) suppressed calcium influx induced by 100 µM vanillin. The TRPV4 channel antagonist (0.1 nM), the function-blocking antibody (ACC-124) (1 µM), and the control IgG antibody (1 µM), when used alone, had no significant effect on [Ca^2+^]ᵢ isolating influx to TRPV4 activation (Fig. 1E and F). Statistical analysis confirmed that exposure to 100 µM vanillin significantly increased calcium peak height in H9 hESCs: an effect that was abolished by the TRPV4 antagonist and function-blocking antibody (Fig. 1G).

TRPV4 channels were also detected in H1 hESCs, appearing as bright green puncta on the cell surface following immunolabeling (Supplementary Fig. S2A and B). Exposure to vanillin (100 µM) induced rapid and significant increases in intracellular calcium in H1 hESCs, as measured by the Fluo-8 assay (Supplementary Fig. S2C). The calcium response to 100 µM was blocked by the TRPV4 antagonist HC067047 (Supplementary Fig. S2C and D).

### Vanillic acid does not induce calcium influx in H9 hESCs

Vanillic acid, a metabolite of vanillin that activates TRPV1 channels, was also investigated as a possible agonist in our experiments. TRPV1 channels were not detectable in hESCs by immunocytochemistry (Supplementary Fig. S3A and B). Consistent with this finding, treatment of H9 hESCs with vanillic acid (100 µM) did not induce a significant increase in intracellular calcium as measured by a Fluo-8 Ca^2+^ assay (Supplementary Fig. S3C).

### Activation of TRPV4 channels with micromolar vanillin alters H9 hESC colony morphology

Colony morphology was significantly changed by 72 h of exposure to 450 µM vanillin (Supplementary Fig. S4). At the end of the incubation, control colonies were round with relatively smooth edges, whereas vanillin caused elongation of colonies and significantly increased the major-to-minor axis ratio (Supplementary Fig. S4A, B, and D). Inclusion of the TRPV4 antagonist in the culture medium prevented this change in aspect ratio (Supplementary Fig. S4C and D).

### Micromolar vanillin inhibits mitochondrial reductases in H9 hESCs through activation of TRPV4 channels

Vanillin was tested in the MTT assay, which measures mitochondrial reductase activity (Ghasemi *et al.*, 2021). Vanillin decreased mitochondrial activity in a concentration-dependent manner with the IC_70_ and IC_50_ values being 221.7 and 402.7 µM, respectively (Supplementary Fig. S5A). H9 hESCs were next pre-incubated with the TRPV4 channel antagonist, followed by exposure to the MTT IC_50_ concentration of vanillin as determined in Supplementary Fig. S5A. The TRPV4 antagonist (0.01–1 nM) prevented loss of mitochondrial activity (Supplementary Fig. S5B); however, higher concentrations of the antagonist (10–1000 nM) were less effective, perhaps because they were cytotoxic (Supplementary Fig. S5B).

### Micromolar vanillin impairs H9 hESC colony growth by inducing detachment and cell death through TRPV4 activation

Features related to colony growth (normalized area and colony detachment) were evaluated using time-lapse imaging over 72 h (Fig. 2A–D). Normalized H9 colony area was extracted from segmented colonies using StemCellQC software (Zahedi *et al.*, 2016). Colony area was defined as the total number of pixels within the segmented colony boundary and was normalized to the initial time point (t = 0). To determine whether vanillin-induced activation of TRPV4 channels affected H9 hESC colony growth, time-lapse data were collected every 4 h. Colony area was significantly decreased in the 136 and 450 µM vanillin groups compared to untreated controls (Fig. 2A), and this reduction was fully rescued by the TRPV4 antagonist (Fig. 2B).

**Figure 2.**
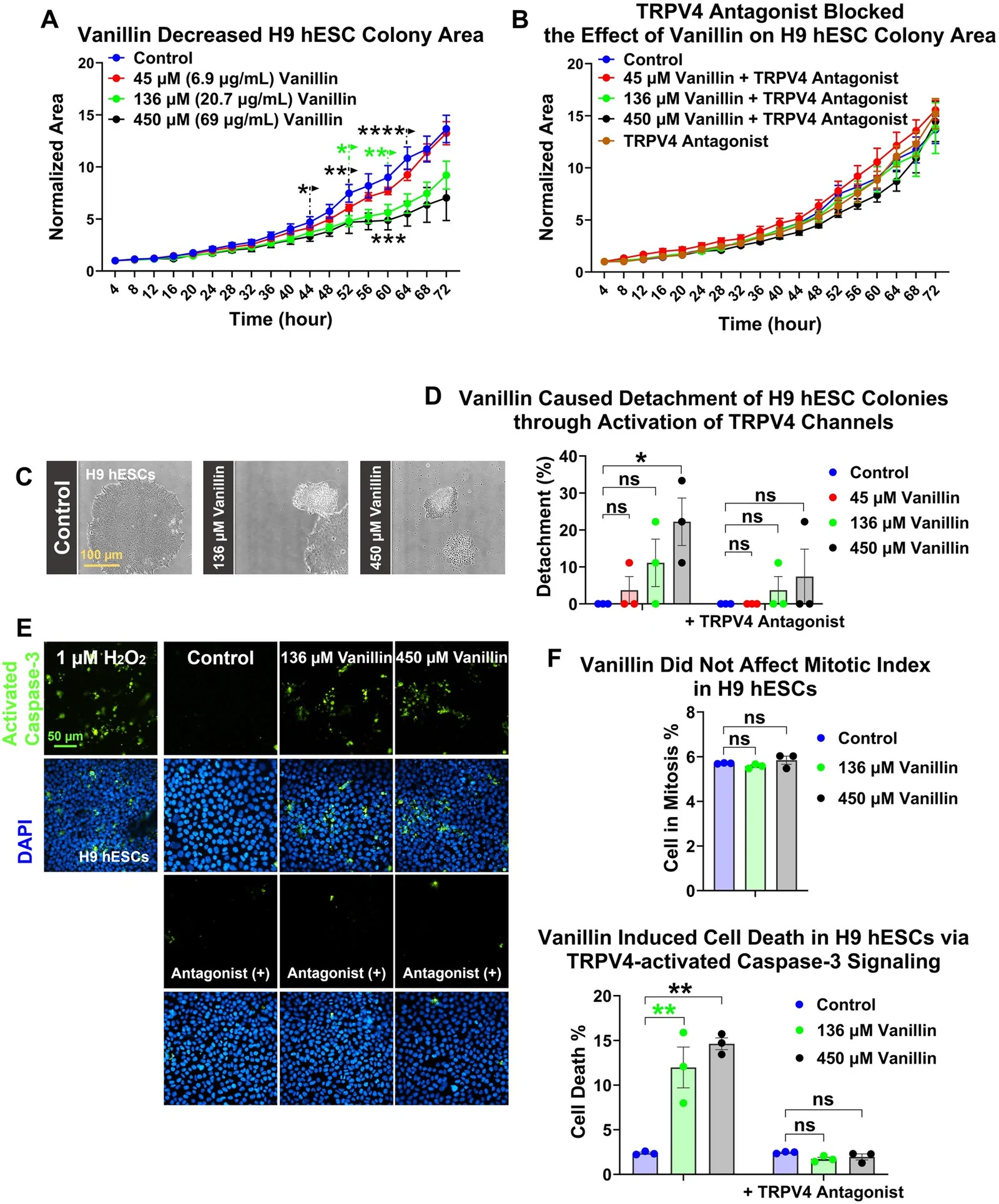
**Vanillin inhibits H9 hESC colony growth by inducing colony detachment and cell death via activation of TRPV4 channels.** (**A**) Time-lapse imaging data showing vanillin (45–450 µM) reduced colony growth (area) over 72 h. (**B**) Vanillin-induced growth inhibition was completely blocked by the TRPV4 antagonist HC067047 (0.1 nM). Data were normalized to the first time point and plotted as means±SEM from three independent experiments, each done with a different passage of cells. In each experiment, three colonies were analyzed/group. Asterisks in Fig. 2A indicate the first time points at which values differed significantly from control, based on a two-way ANOVA followed by Dunnett’s *post hoc* test. *P < 0.05, **P < 0.01, ***P < 0.001, ****P < 0.0001. (**C**) Phase-contrast images of hESC colonies at the final time point in control, 136 µM vanillin and 450 µM vanillin groups, showing colony rounding and detachment in treated groups. Scale bar = 100 µm. (**D**) Quantification of colony detachment in control, vanillin-treated, and vanillin + the TRPV4 antagonist (0.1 nM HC067047) groups. Bars are plotted as means±SEM from three independent experiments, each done with a different passage of cells. In each experiment, three colonies were analyzed/group. (**E**) Immunofluorescence micrographs of hESCs treated with vanillin, vanillin + the TRPV4 antagonist (0.1 nM HC067047), or H_2_O_2_ (positive control) and labeled with an antibody against activated caspase-3 (green) to detect apoptosis. Merged micrographs include DAPI counterstaining (blue). Scale bar = 50 µm. Activated caspase-3 labeling was increased with vanillin or H_2_O_2_ but absent in the presence of the TRPV4 antagonist HC067047 (0.1 nM). (**F**, top) Quantification of the mitotic index (metaphase + anaphase) excluding apoptotic (activated caspase-3 positive) cells showed that vanillin did not affect mitosis. (**F**, bottom) Percentage of cell death (activated caspase-3-positive) following treatment with vanillin±TRPV4 antagonist (0.1 nM HC067047). In (F), three independent experiments were done, each with a different passage of cells. In each experiment, three colonies/group were analyzed, and 100 cells were counted/colony. Statistical analysis was performed using one-way ANOVA followed by Dunnett’s *post hoc* test with treated groups compared to the control. ns = non-significant, *P < 0.05, **P < 0.01.

H9 colony detachment was quantified manually from phase-contrast time-lapse images. Only colonies that remained in the field of view throughout the incubation period were included in the analysis (Fig. 2C). Vanillin caused a concentration-dependent increase in colony detachment (Fig. 2C), which was significant at 450 µM, and was prevented by the TRPV4 antagonism (Fig. 2D).

To determine whether vanillin impaired H9 colony growth by inhibiting cell proliferation, the mitotic index was scored in cells exposed to 136 and 450 µM vanillin. Samples were co-labeled with an antibody to activated caspase-3 to exclude apoptotic cells from the analysis (Fig. 2E). Across control and treated groups, the mitotic index remained around 5.5% with no significant differences, indicating that vanillin did not alter cell proliferation (Fig. 2F). Similar to H9 colonies, the mitotic index did not differ significantly between H1 control and treated groups, indicating that vanillin did not alter cell proliferation in the H1 line (Supplementary Fig. S6B).

H9 and H1 hESC colonies treated with vanillin for 72 h were evaluated for cell death using immunolabeling with an activated caspase-3 antibody. Antibody signal was significantly increased in both the positive control group (H_2_O_2_) and groups treated with 136 and 450 µM vanillin, indicating elevated apoptosis (Fig. 2E, Supplementary Fig. S6A). In contrast, no activated caspase-3 antibody binding was observed when vanillin treatments included the TRPV4 antagonist. Quantitative analysis confirmed that vanillin significantly increased the number of caspase-3-positive (dead) cells: an effect that was prevented by co-treatment with the TRPV4 antagonist (Fig. 2F, Supplementary Fig. S6C).

Vanillin-induced cell death was further quantified using the brightness/total area ratio assay in StemCellQC software, which measures the proportion of bright pixels (characteristic of dead cells) relative to total colony area (Zahedi *et al.*, 2016). Phase-contrast micrographs showed dead cells (pseudocolored yellow) localized on top of H9 hESC colonies (Supplementary Fig. S7A). Treatment with 136 and 450 µM vanillin for 72 h resulted in a significant increase in the brightness/total area ratio compared with controls (Supplementary Fig. S7B). This increase was significantly attenuated by co-treatment with the TRPV4 channel antagonist (Supplementary Fig. S7B).

### Nanomolar and micromolar vanillin increases cell-to-cell gaps and decreases EpCAM expression in H9 hESCs by activating TRPV4 channels

In control colonies, cells were tightly packed, and gaps did not appear between adjacent cells. However, gaps formed between cells following exposure to 0.45 and 45 µM vanillin (Fig. 3A). To test the hypothesis that gap formation occurred because expression of a cell-to-cell adhesion molecule (EpCAM) decreased, colonies were immunolabeled with an anti-EpCAM antibody. Untreated colonies displayed strong EpCAM expression around the periphery of each cell (Fig. 3B). After 3 days of exposure to vanillin, EpCAM expression was significantly reduced, as evident in both whole-colony and enlarged micrographs (Fig. 3B). Quantitative analysis confirmed that both line area/dot area ratio and the mean fluorescence intensity of EpCAM labeling were significantly decreased following vanillin exposure (Fig. 3C and D).

**Figure 3.**
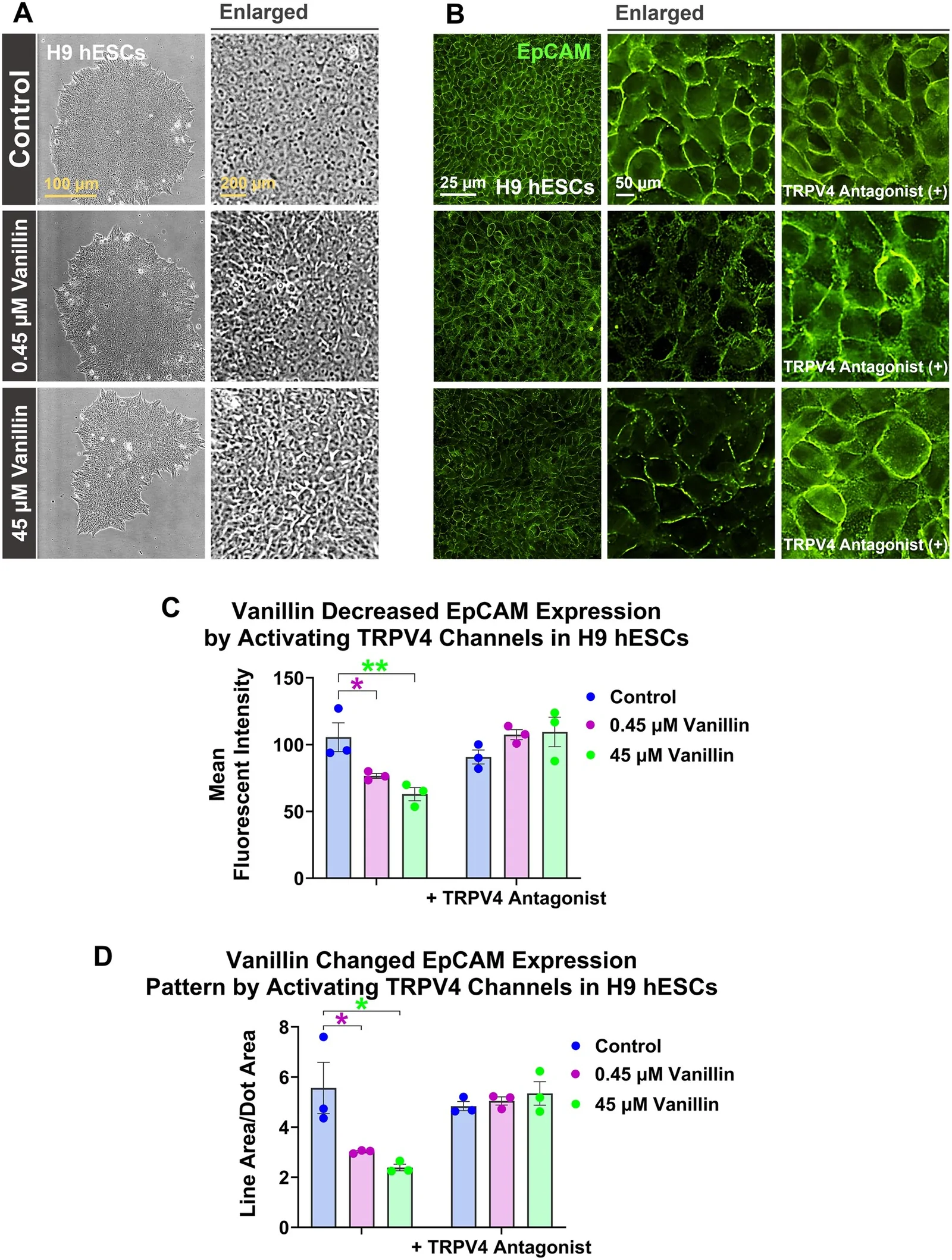
**Vanillin increases intercellular gaps and reduces EpCAM expression in H9 hESC colonies by activation of TRPV4 channels.** (**A**) Phase-contrast images of H9 hESC colonies treated with vanillin for 72 h. An increase in intercellular gaps (bright regions) was observed in all vanillin-treated groups compared to the control. Scale bars = 100 µm (low magnification) and 200 µm (high magnification). (**B**) Immunofluorescence micrographs of control hESC colonies and colonies treated with vanillin and with vanillin + the TRPV4 antagonist (0.1 nM HC067047) stained with EpCAM antibody (green). Scale bars = 25 µm (low magnification) and 50 µm (high magnification). (**C, D**) Quantification of EpCAM labeling in untreated and treated colonies. Mean fluorescence intensity (C) and line area/dot area ratio (D) were significantly decreased in vanillin-treated colonies. Co-treatment with the TRPV4 antagonist HC067047 (0.1 nM) prevented these changes. Data are presented as means±SEM from three independent experiments, each with a different passage of cells. In each experiment, three colonies/group were analyzed, and 100 cells were counted/colony. Statistical analysis was performed using a one-way ANOVA followed by Dunnett’s *post hoc* test with treated groups compared to the control. *P < 0.05, **P < 0.01.

Inclusion of the TRPV4 antagonist effectively prevented the loss of EpCAM expression in the treated groups (Fig. 3B). In the presence of the antagonist, EpCAM levels and localization remained comparable to controls, with no significant changes in the expression pattern or mean fluorescence intensity (Fig. 3C and D). The TRPV4 channel antagonist alone had no significant effect on EpCAM expression at the cell surface (Fig. 3B–D).

### Nanomolar vanillin induces loss of pluripotency and endodermal marker expression in H9 hESCs by activating TRPV4 channels

In previous studies, EpCAM was expressed in pluripotent but not differentiated hESCs (Lu *et al.*, 2010; Ng *et al.*, 2010) and the formation of gaps in hESC colonies was associated with the loss of pluripotency (Lin *et al.*, 2021). To determine whether vanillin-induced reduction in EpCAM and increased in gap formation correlated with loss of pluripotency, we examined the expression of the pluripotency marker OCT4 and three germ layer markers after 6 days of exposure to 0.45 µM vanillin (Fig. 4). Untreated control colonies exhibiting strong, uniform nuclear OCT4 staining were used as the reference for pluripotency maintenance (Fig. 4A). OCT4 expression was significantly reduced in colonies treated with 0.45 µM vanillin. Co-treatment with the TRPV4 antagonist prevented this reduction and maintained OCT4 expression at levels comparable to controls (Fig. 4A). Quantification analysis confirmed significant loss of OCT4 in the vanillin group and rescue by the TRPV4 antagonist (Fig. 4B).

**Figure 4.**
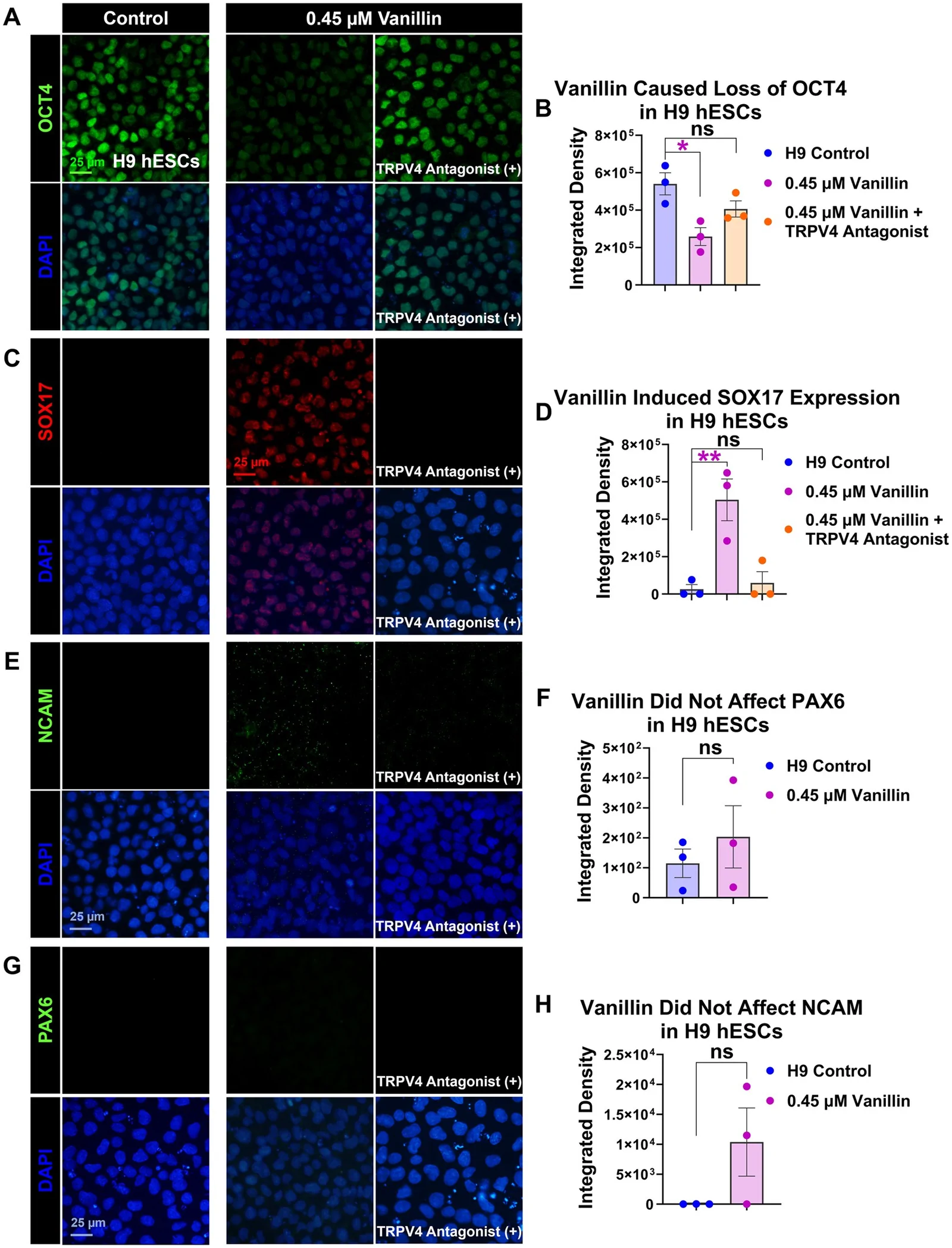
**Activation of TRPV4 channels by vanillin induces loss of pluripotency and expression of the endodermal marker SOX17 in H9 hESCs.** (**A**) Immunofluorescence micrographs showing reduced OCT4 expression (green) after 6 days of exposure to 0.45 µM vanillin. Co-treatment with the TRPV4 antagonist HC067047 (0.1 nM) preserved OCT4 expression. (**B**) Quantification of OCT4 expression levels in control and vanillin ± the TRPV4 antagonist groups. (**C**) Representative immunostaining for SOX17 (red) after 6 days of vanillin exposure, showing upregulation in hESCs treated with 0.450 µM vanillin. Co-treatment with the TRPV4 antagonist HC067047 (0.1 nM) prevented SOX17 induction. (**D**) Quantification of SOX17 expression in control and vanillin ± the TRPV4 antagonist groups. (**E–H**) Representative immunostaining for NCAM (green, E) and PAX6 (green, G) after 6 days of treatment showed low, non-significant upregulation in response to 0.45 µM vanillin (F, H), which was attenuated by TRPV4 antagonist HC067047 (0.1 nM). All micrographs (A, C, E, G) were merged with DAPI (blue) to visualize nuclei. Scale bar = 25 µm. Data are presented as means±SEM from three independent experiments, each with a different passage of cells. In each experiment, three colonies/group were analyzed, and 100 cells were counted/colony. Statistical analysis was performed using one-way ANOVA followed by Dunnett’s *post hoc* test with treated groups compared to the control. ns = non-significant, *P < 0.05, **P < 0.01.

Immunostaining for the endodermal marker SOX17 revealed no signal in control colonies, as expected for undifferentiated hESCs (Fig. 4C). However, strong nuclear SOX17 expression was observed in colonies treated with 0.45 µM vanillin, consistent with endodermal induction (Fig. 4C). This effect was fully blocked by the TRPV4 antagonist (Fig. 4D). Expression levels of ectodermal and mesodermal markers were not significantly altered under these conditions (Fig. 4E–H).

### Activation of TRPV4 channels by nanomolar concentrations of vanillin

Vanillin at 450 nM (0.45 μM) caused an exit from pluripotency and expression of the endodermal marker SOX17 (Fig. 4). Since our prior work on calcium influx used only micromolar concentrations of vanillin (Fig. 1), we followed up to determine if a nanomolar concentration could induce calcium influx via TRPV4 (Fig. 5). Nanomolar concentrations (450 nm) of vanillin induced a rapid and significant increase in intracellular calcium in H9 hESCs, peaking at approximately 3000 RFU within the first minute of exposure (Fig. 5A). This response was blocked by co-treatment with either a TRPV4 antagonist or a TRPV4-blocking antibody, which both reduced intracellular calcium levels to values comparable to the HHBS buffer control (Fig. 5B). A similar response was observed in H1 hESCs treated with 450 nM vanillin (Supplementary Fig. S2E and F).

**Figure 5.**
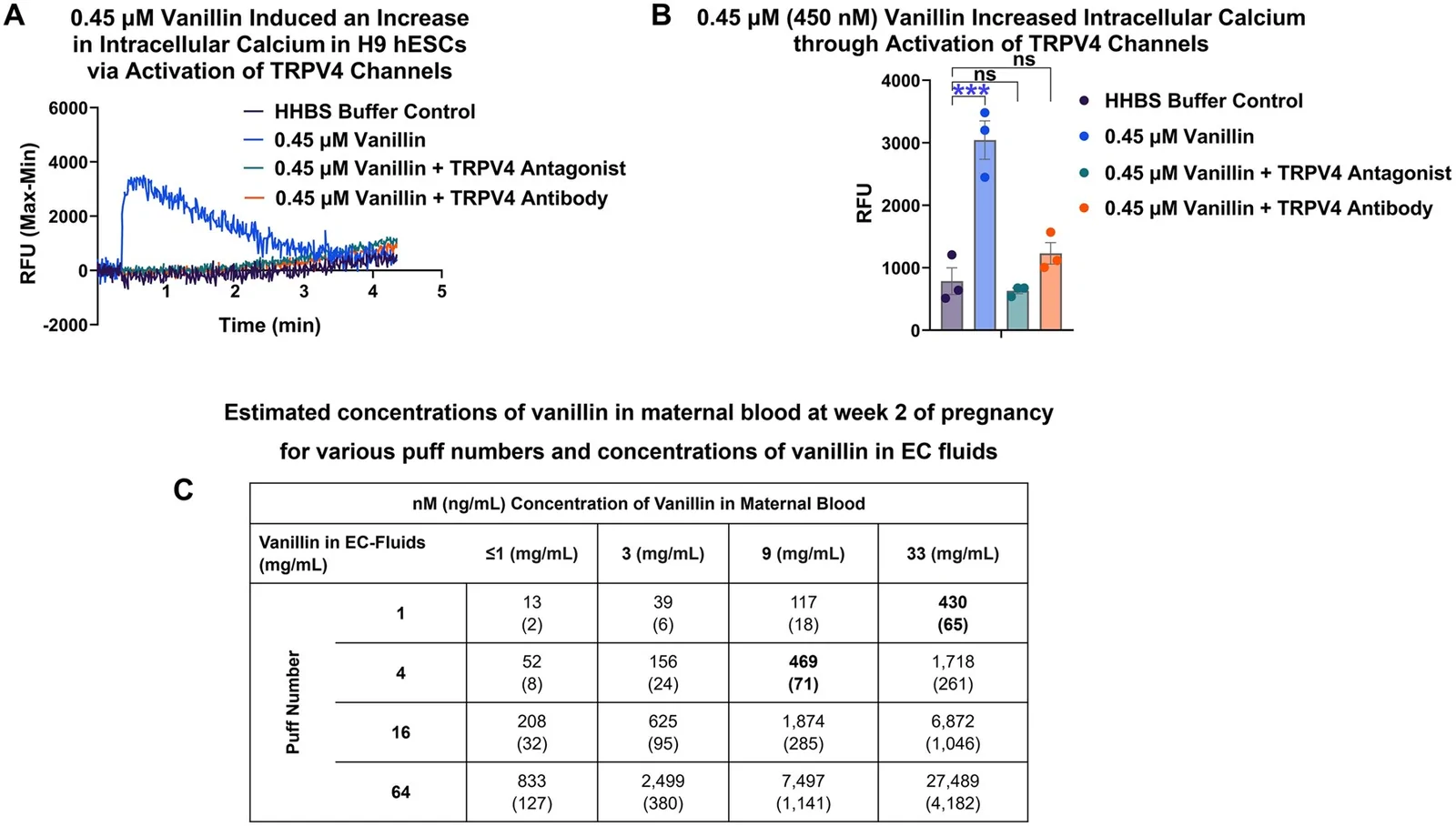
**Nanomolar vanillin concentrations relevant to embryonic exposure induce calcium influx in H9 hESCs by activation of TRPV4 channels.** (**A**) Representative graph showing increased intracellular calcium levels in H9 hESCs following exposure to 0.45 µM vanillin. The response was inhibited by the TRPV4 antagonist HC067047 (0.1 nM) and a TRPV4-blocking antibody ACC-124, (1 μM). RFU (relative fluorescence units) values were baseline-subtracted. (**B**) Quantification of peak calcium responses for HHBS buffer control, vanillin, and vanillin plus the TRPV4 antagonist and antibody. Bars represent the means±SEM from three independent experiments, each done with a different passage of cells. Statistical analysis was performed using one-way ANOVA followed by Dunnett’s *post hoc* test compared to the control. ns = non-significant, ***P < 0.001. (**C**) Predicted maternal blood concentrations of vanillin for varying electronic cigarette (EC) fluid concentrations and puff numbers per hour. Values are shown as nM, with ng/ml in parentheses. Blue-highlighted cells indicate exposure scenarios yielding blood concentrations comparable to those used in this study (430–469 nM; 65–71 ng/ml).

### Estimation of vanillin concentrations in maternal blood at Weeks 2–3 of pregnancy

To estimate vanillin concentrations in the maternal circulation during EC use, we applied the quantitative inhalation exposure model described in the Materials and methods section, using a range of vanillin concentrations reported in commercial EC refill fluids (≤1–33 mg/ml) (Tierney *et al.*, 2016; Behar *et al.*, 2018) and a range of puff rates (1–64 puffs/h). Predicted vanillin concentrations in maternal blood at Week 2 of pregnancy spanned the nanomolar to low micromolar range, from 13 nM at the lowest exposure scenario (1 puffs/h; ≤1 mg/ml refill fluid) to 27 489 nM at the highest (64 puffs/h; 33 mg/ml refill fluid) (Fig. 5C). For exposure scenarios reflecting more typical EC use patterns, 1 or 4 puffs/h combined with refill fluid concentrations of 9 or 33 mg/ml, predicted maternal blood concentrations were close to 450 nM (highlighted cells, Fig. 5C): the vanillin concentration that activated TRPV4-mediated calcium influx and the downstream cascade leading to endodermal differentiation.

## Discussion

The central finding of this study is that vanillin, one of the most prevalent flavor chemicals in commercial EC refill fluids, disrupts pluripotency and cell fate specification in hESCs through two concentration-dependent mechanisms: cytotoxic damage at micromolar concentrations and aberrant cell fate specification at nanomolar concentrations (Fig. 6). Both effects are mediated through activation of TRPV4, a calcium-permeable ion channel expressed in hESCs, and the cell fate effects occur within a concentration range that our exposure model predicts are achievable in the maternal circulation, and by extension the embryo, during EC use in pregnancy. These findings, obtained with hESCs as a model of the epiblast, raise the possibility that vanillin may similarly disrupt early human embryonic development *in vivo*, as discussed below.

**Figure 6.**
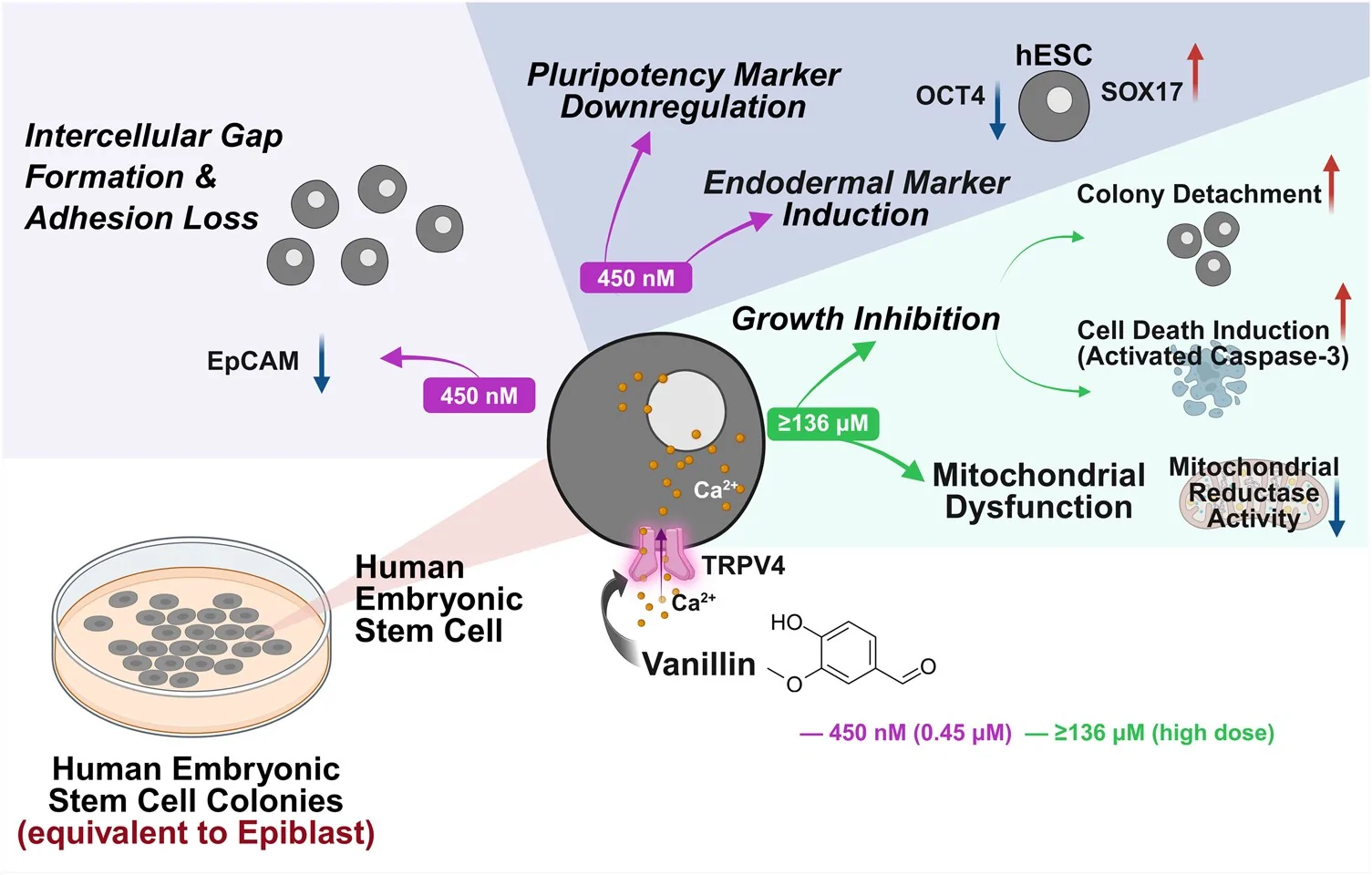
**Summary of the main results of the study.** Created in BioRender. Etemadi, S. (2026) https://BioRender.com/sz2o5ha.

Micromolar and nanomolar concentrations of vanillin affected different endpoints. This difference in endpoint sensitivity could be due to the larger amount of calcium influx that occurred at micromolar concentrations (peak RFU = 5000 ± 500) versus nanomolar concentrations (peak RFU = 2500 ± 200). At micromolar concentrations, vanillin induced a suite of cytotoxic responses in hESCs, including reduced colony growth, increased cell death, cell detachment, morphological disruption and impaired mitochondrial function. These effects were fully prevented by TRPV4 antagonism, establishing TRPV4-mediated calcium influx as the upstream driver of this toxicity. The pattern is consistent with reported pro-apoptotic activity for vanillin in cancer cell lines, where it activates caspase-3/8 pathways and suppresses pro-survival Akt-mediated signaling such as Wnt/β-catenin across a comparable micromolar concentration range (Srinual *et al.*, 2017; Mai *et al.*, 2018; Yousuf *et al.*, 2021). Mitochondrial dysfunction and reactive oxygen species (ROS) generation have similarly been implicated in vanillin-mediated cytotoxicity in oncology models (Kim *et al.*, 2014; Naz *et al.*, 2018; Saibabu *et al.*, 2021), and hESCs share key similarities with cancer cells, including rapid proliferation and sensitivity to oxidative stress (Dayem *et al.*, 2010; Shay and Wright, 2010; Günes and Rudolph, 2013). Although contributions from caspase-independent or parallel stress pathways cannot be excluded, the near-complete rescue of hESCs by TRPV4 antagonism indicates that TRPV4 signaling is the predominant upstream driver of micromolar vanillin toxicity. In the embryo, such effects could reduce the size of the epiblast cell pool, impair gastrulation, or result in embryo loss.

The more striking and developmentally significant finding, however, is the biological activity at a nanomolar concentration of vanillin, which is orders of magnitude lower than those required to produce overt cytotoxicity. At 450 nM (0.45 µM), vanillin activated TRPV4-mediated calcium influx, downregulated the pluripotency markers EpCAM and OCT4, induced gap formation in hESC colonies, and drove differentiation toward endoderm, as evidenced by elevated SOX17 expression in the absence of ectodermal (PAX6) or mesodermal (NCAM) markers. SOX17 induction without activation of alternative lineage markers, supports a directed transition toward endoderm rather than non-specific loss of cell identity (D’Amour *et al.*, 2005; Teo *et al.*, 2011); these points could be investigated in future studies using additional germ layer markers. The complete prevention of SOX17 expression by TRPV4 antagonism confirms that this differentiation response is TRPV4-dependent. These findings are consistent with a growing body of evidence that TRPV4-mediated calcium signaling regulates stem cell fate decisions across multiple lineages and cell types (Adapala *et al.*, 2013; Willard *et al.*, 2021; Chen *et al.*, 2024; Tytti *et al.*, 2024) and extend that role to endodermal gene expression in human pluripotent cells.

Critically, the 450 nM concentration at which these developmental effects occur falls squarely within the range of vanillin concentrations that our quantitative inhalation exposure model predicts will reach maternal blood during typical EC use. For common use patterns (1–4 puffs per hour with refill fluid vanillin concentrations of 9–33 mg/ml) predicted maternal blood concentrations are similar to 450 nM. Moreover, inhaled nicotine reaches the fetal circulation at nanomolar concentrations (3–154 nM) (Luck *et al.*, 1985; Suter and Aagaard, 2020), overlapping with our model predictions and supporting the biological plausibility of embryonic vanillin exposure at developmentally active levels. The higher micromolar concentrations tested in this study were included to define the full dose–response landscape, and some of the micromolar concentrations we used may exceed *in vivo* concentrations. It should be noted however, our model does not predict cumulative exposures over days, weeks, months, or years, and chronic exposures may be higher than those reported in Fig. 5C, in which case both cytotoxicity and endodermal differentiation may be factors *in vivo*. Because there is no regulatory requirement to disclose flavoring ingredients or concentrations on EC packaging, pregnant women using sweet, fruity, dessert, or certain tobacco-flavored products may be unknowingly inhaling vanillin at concentrations sufficient to perturb embryonic cell fate.

The developmental window implicated here, Weeks 2–3 of pregnancy, when gastrulation and germ layer specification are underway, is one during which many women do not yet know they are pregnant. Unscheduled SOX17 induction in epiblast cells, consistent with endodermal specification, at this stage could disrupt the coordinated cell movements and signaling events required for proper embryonic patterning, potentially contributing to outcomes such as embryo loss or growth restriction. Epidemiological studies have reported increased rates of stillbirth and small-for-gestational-age births in women who vaped during pregnancy (Regan *et al.*, 2021; Shittu *et al.*, 2022; Deprato *et al.*, 2025), and while such studies cannot resolve effects at this early developmental stage, our *in vitro* model provides mechanistic insight into a previously inaccessible window of human development. The effects of vanillin on germ layer specification that we observed would likely be embryo lethal and would not be picked up in epidemiological studies that look at birth endpoints.

## Conclusions

Taken together, these findings establish vanillin as a biologically active EC constituent with a dual mechanism of developmental toxicity: cytotoxic at micromolar concentrations and pro-differentiation at nanomolar concentrations, with both effects operating through TRPV4-mediated calcium signaling. The nanomolar activity is particularly concerning given its overlap with predicted *in vivo* exposure levels. These data support the conclusion that EC products containing vanillin pose meaningful risks to embryonic health during the earliest stages of pregnancy. The vanillin data align well with our prior findings demonstrating that nanomolar to micromolar concentrations of menthol and WS-23 (a synthetic coolant), chemicals frequently used at high concentrations in ECs (Behar *et al.*, 2018; Wong *et al.*, 2023), activate TRPA1 and TRPM8 in hESCs (Etemadi and Talbot, 2025; Etemadi *et al.*, 2025). Collectively, these data on vanillin, menthol and WS-23 show that hESC-expressed TRP channels are sensitive to multiple EC-relevant chemicals, raising concern that such exposures could compromise early developmental events such as germ layer differentiation and gastrulation, with potential adverse outcomes and reinforcing the need for ingredient disclosure on EC packaging and clinical counselling of patients who vape during or around pregnancy.

## Supplementary Material

deag126_Supplementary_Materials_and_Methods

deag126_Supplementary_Figure_S1

deag126_Supplementary_Figure_S2

deag126_Supplementary_Figure_S3

deag126_Supplementary_Figure_S4

deag126_Supplementary_Figure_S5

deag126_Supplementary_Figure_S6

deag126_Supplementary_Figure_S7

deag126_Supplementary_Table_S1

## Data Availability

The data underlying this article are available in the article and in its online supplementary material.
