# Supplementary material for: Nanomolar vanillin, an e-cigarette flavorant, appears to disrupt pluripotency and promote endodermal differentiation in human embryonic stem cells via TRPV4 activation
Source: Hum Reprod. 2026 Aug 12;41(10):1688–702. doi: 10.1093/humrep/deag126 (PMC13635455; doi:10.1093/humrep/deag126)
Supplement: Supplementary file 1 [file deag126_supplementary_figure_s1.pdf]

Human Reproduction, 2026, 1–1
https://doi.org/10.1093/humrep/deag126
Supplementary Data

human reproduction
OXFORD

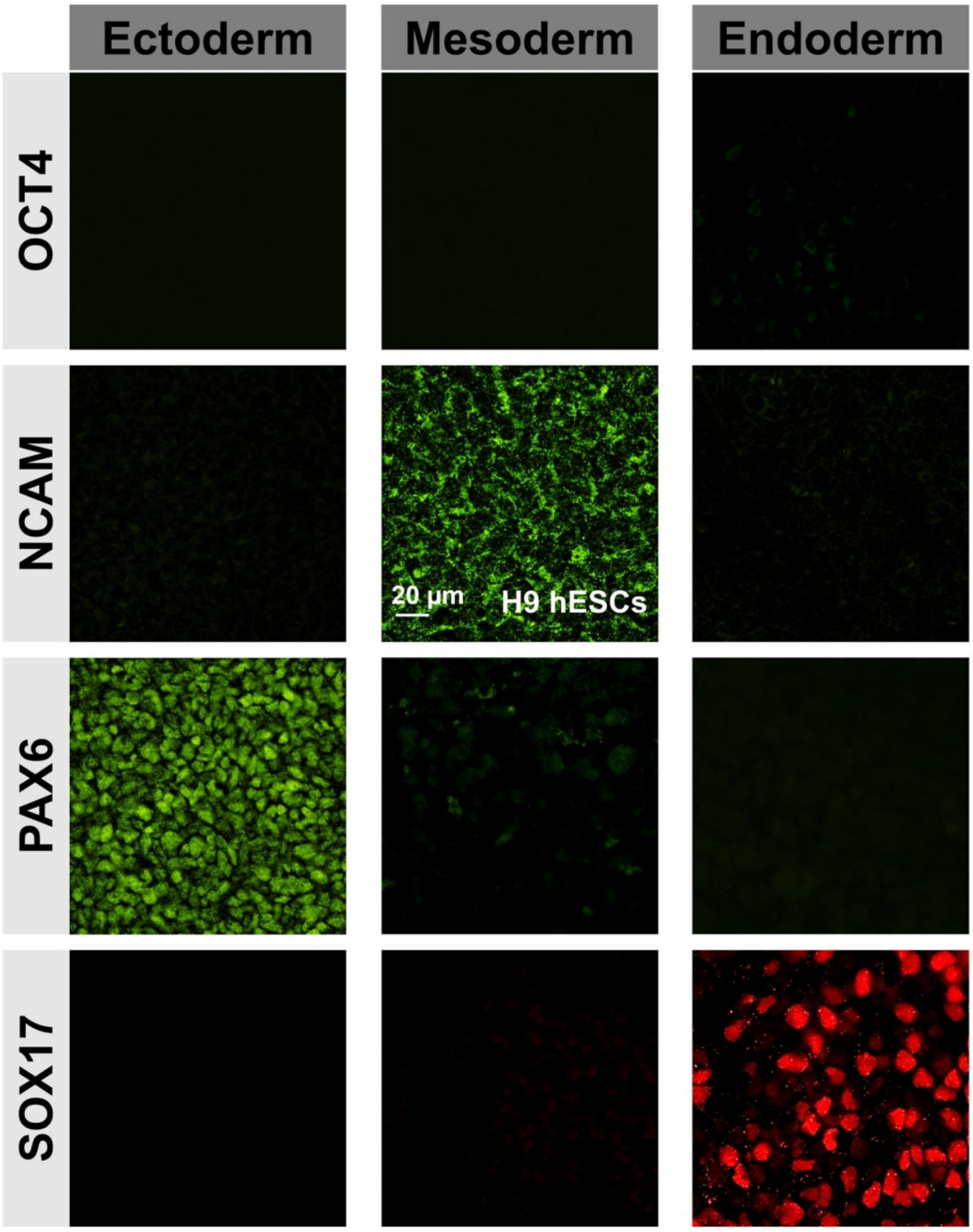

Supplementary Figure S1. Marker expression of germ layers and pluripotency in differentiated H9 hESCs. Representative micrographs showing germ layers labeled with antibodies specific to mesoderm (NCAM), ectoderm (PAX6), and endoderm (SOX17), and the pluripotency marker OCT4. Scale bar = 20 μm. Immunolabeling was performed in three independent experiments, each done with a different passage of cells.
