# Supplementary material for: Nanomolar vanillin, an e-cigarette flavorant, appears to disrupt pluripotency and promote endodermal differentiation in human embryonic stem cells via TRPV4 activation
Source: Hum Reprod. 2026 Aug 12;41(10):1688–702. doi: 10.1093/humrep/deag126 (PMC13635455; doi:10.1093/humrep/deag126)
Supplement: Supplementary file 2 [file deag126_supplementary_figure_s2.pdf]

Human Reproduction, 2026, 1–1
https://doi.org/10.1093/humrep/deag126
Supplementary Data

human reproduction
OXFORD

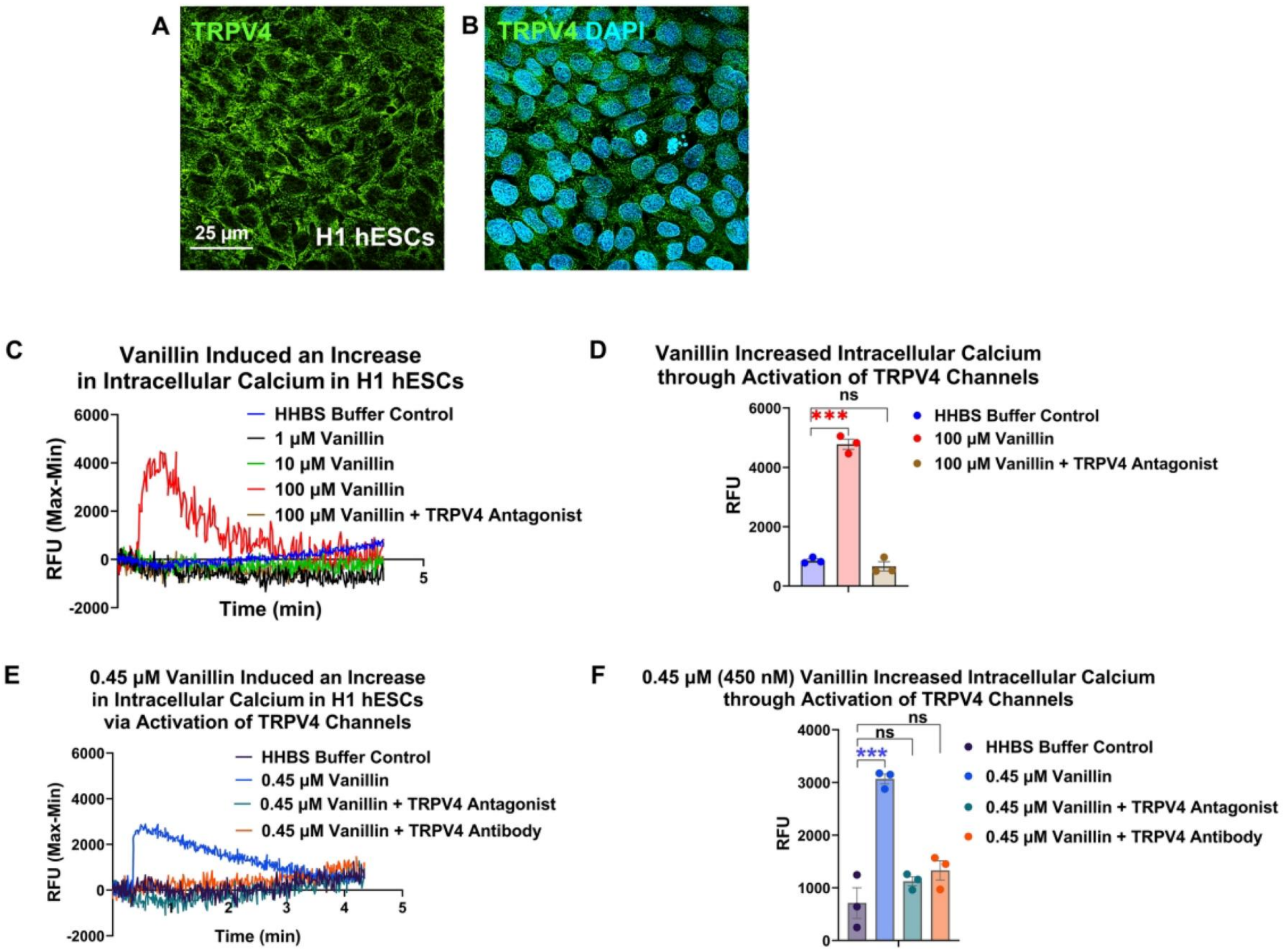

Supplementary Figure S2. Vanillin induces calcium influx in H1 hESCs. (**A**, **B**) Representative immunofluorescence micrographs of H1 hESCs labeled with a human TRPV4 antibody (green) (A) and counterstained with DAPI to visualize nuclei (blue) (B). Scale bar = 25 μm. Immunolabeling was performed in three independent experiments, each done with a different passage of cells. (**C**) Representative graph showing increased intracellular calcium levels in H1 hESCs following exposure to 100 μM vanillin. Calcium influx induced by vanillin was blocked by the TRPV4 antagonist HC067047 (0.1 nM), which itself had no effect on intracellular calcium levels. RFU (relative fluorescence units) for each graph is baseline subtracted. (**D**) Quantification of peak calcium responses for HHBS buffer control, vanillin, and vanillin plus the TRPV4 antagonist. (**E**) Representative graph showing increased intracellular calcium levels in H1 hESCs following exposure to 0.45 μM vanillin. Calcium influx induced by vanillin was blocked by both the TRPV4 antagonist (0.1 nM HC067047) and the TRPV4 function-blocking antibody (1 μM ACC-124). RFU (relative fluorescence units) for each graph is baseline subtracted. (**F**) Quantification of peak calcium responses for HHBS buffer control, vanillin, and vanillin plus the TRPV4 antagonist and antibody. Bars represent the means±SEM from three independent experiments, each done with a different passage of cells. Statistical analysis was performed using one-way ANOVA followed by Dunnett's *post hoc* test compared to the control. ns = non-significant, $^{***}P < 0.001$.
