# Supplementary material for: Nanomolar vanillin, an e-cigarette flavorant, appears to disrupt pluripotency and promote endodermal differentiation in human embryonic stem cells via TRPV4 activation
Source: Hum Reprod. 2026 Aug 12;41(10):1688–702. doi: 10.1093/humrep/deag126 (PMC13635455; doi:10.1093/humrep/deag126)
Supplement: Supplementary file 3 [file deag126_supplementary_figure_s3.pdf]

Human Reproduction, 2026, 1–1
https://doi.org/10.1093/humrep/deag126
Supplementary Data

human reproduction
OXFORD

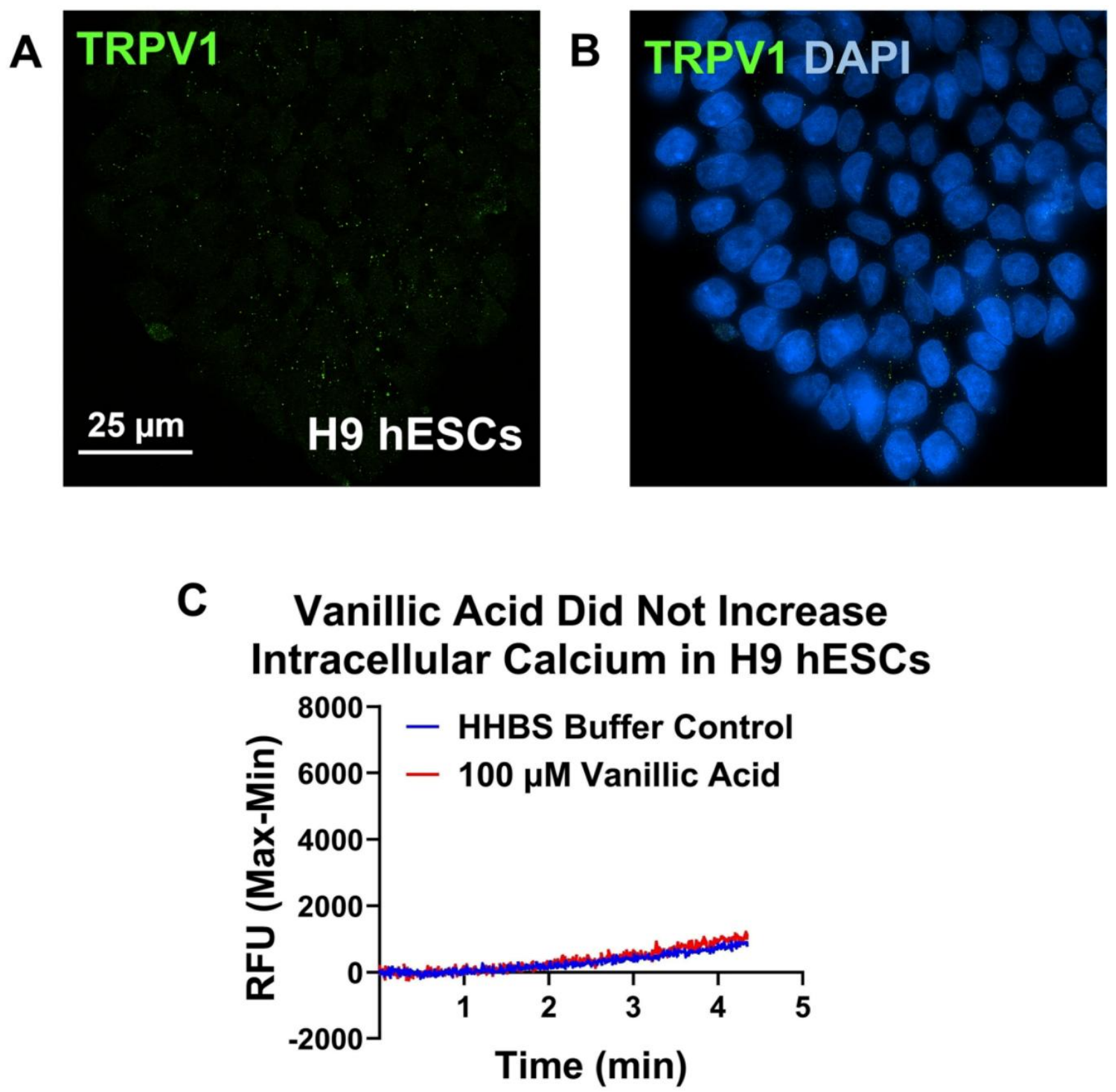

Supplementary Figure S3. Vanillic acid does not induce calcium influx in H9 hESCs. (**A, B**) Representative immunofluorescence micrographs of H9 hESCs labeled with a human TRPV1 antibody (green) (A) and counterstained with DAPI to visualize nuclei (blue) (B). Scale bar = 25 μm. Immunolabeling was performed in three independent experiments, each done with a different passage of cells. (**C**) Representative graph showing no significant increase in intracellular calcium levels in hESCs following exposure to 100 μM vanillic acid compared to HHBS buffer control. RFU (relative fluorescence units) for each graph is baseline subtracted. Three independent experiments were performed using different passages.
