# Supplementary material for: Nanomolar vanillin, an e-cigarette flavorant, appears to disrupt pluripotency and promote endodermal differentiation in human embryonic stem cells via TRPV4 activation
Source: Hum Reprod. 2026 Aug 12;41(10):1688–702. doi: 10.1093/humrep/deag126 (PMC13635455; doi:10.1093/humrep/deag126)
Supplement: Supplementary file 4 [file deag126_supplementary_figure_s4.pdf]

Human Reproduction, 2026, 1–1
https://doi.org/10.1093/humrep/deag126
Supplementary Data

human reproduction

OXFORD

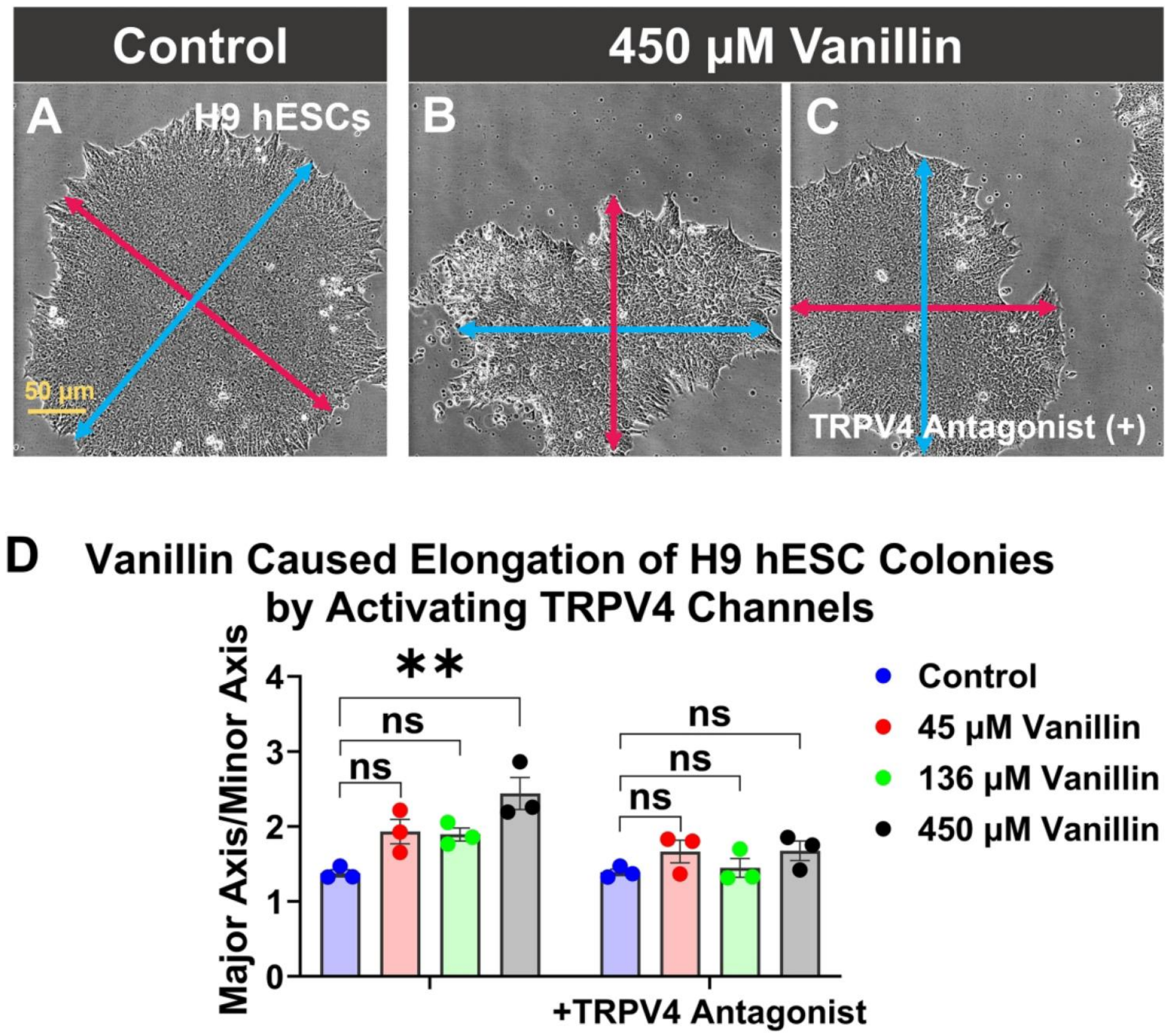

Supplementary Figure S4. Vanillin induces H9 hESC colony elongation by activation of TRPV4 channels. (**A–C**) Representative phase-contrast images of hESC colonies at the 72-h time point showing major (red arrows) and minor (blue arrows) axes in control (A), 450 μM vanillin (B), and 450 μM vanillin + the TRPV4 antagonist (C) groups. Scale bar = 50 μm. (**D**) Quantification of colony shape by major-to-minor axis ratio after 72 h of treatment. Vanillin significantly increased colony elongation: an effect that was prevented by co-treatment with the TRPV4 antagonist HC067047 (0.1 nM). Data are presented as means±SEM from three independent experiments, each done with a different passage of cells. In each experiment, three colonies were analyzed per group. Statistical analysis was performed using one-way ANOVA followed by Dunnett's *post hoc* test compared to the control. ns = non-significant, $^{**}P < 0.01$.
