# Supplementary material for: Nanomolar vanillin, an e-cigarette flavorant, appears to disrupt pluripotency and promote endodermal differentiation in human embryonic stem cells via TRPV4 activation
Source: Hum Reprod. 2026 Aug 12;41(10):1688–702. doi: 10.1093/humrep/deag126 (PMC13635455; doi:10.1093/humrep/deag126)
Supplement: Supplementary file 5 [file deag126_supplementary_figure_s5.pdf]

Human Reproduction, 2026, 1–1
https://doi.org/10.1093/humrep/deag126
Supplementary Data

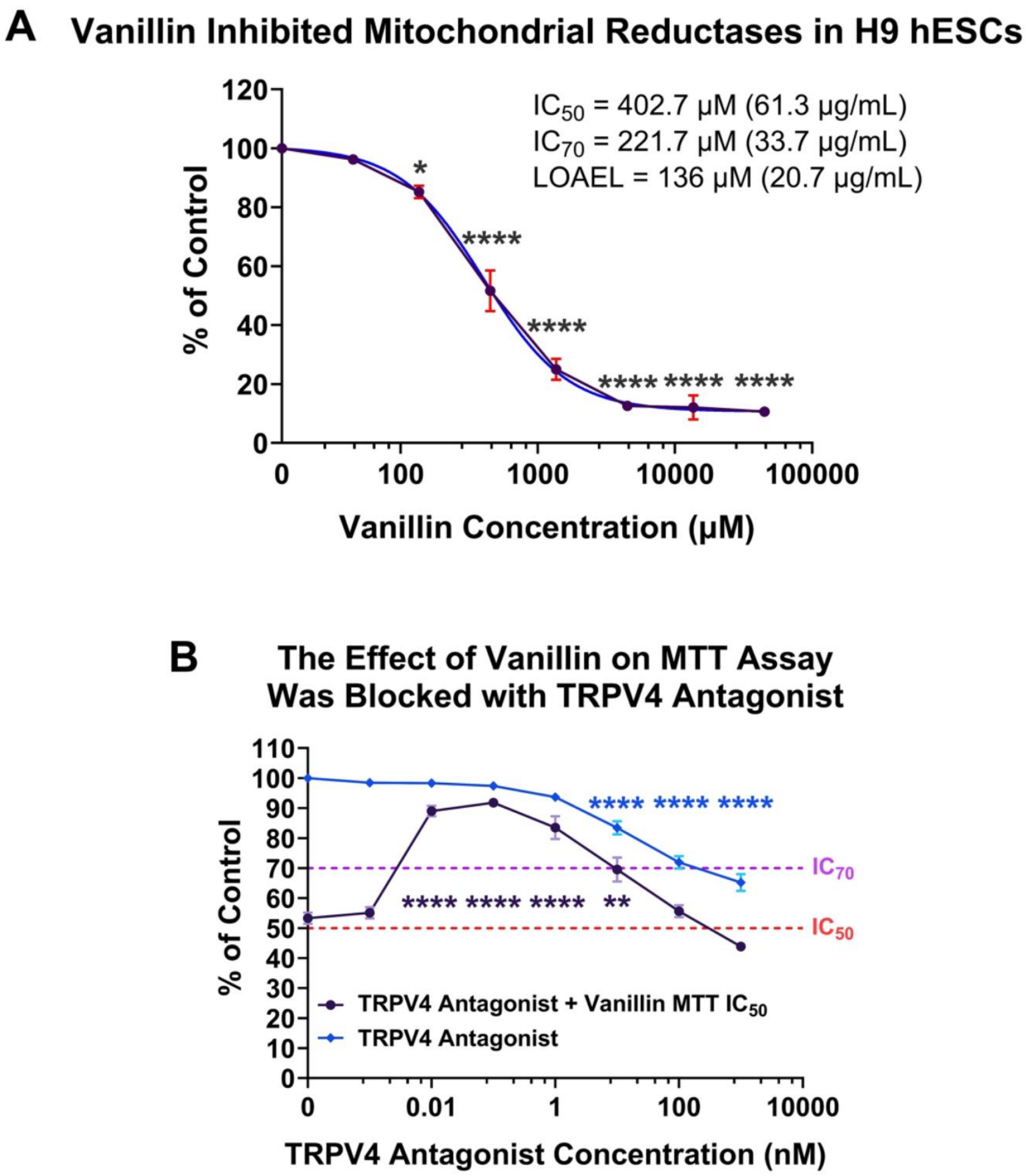

Supplementary Figure S5. Vanillin reduces mitochondrial reductase activity in H9 hESCs by activation of TRPV4 channels. (**A**) Concentration–response curve for vanillin obtained from the MTT assay. Absorbance values were normalized to the control and plotted as a function of vanillin concentration. The IC_{50}, IC_{70}, and lowest observed adverse effect level (LOAEL) were determined as described in the Materials and methods section. (**B**) MTT assay concentration–response curve in the presence of the TRPV4 antagonist HC067047. Co-treatment with HC067047 (0.1 nM) blocked vanillin-induced reductions in mitochondrial activity. Higher concentrations of HC067047 (10–1000 nM) alone significantly affected the MTT assay. Red and purple dashed lines indicate the IC_{50} and IC_{70} values for vanillin, respectively. Data are presented as means±SEM from three independent experiments, each done with a different passage of cells. Statistical analysis was performed using one-way ANOVA followed by Dunnett's *post hoc* test compared to control or the lowest vanillin concentration tested. *$P < 0.05$, **$P < 0.01$, ***$P < 0.001$, ****$P < 0.00001$.
