# Supplementary material for: Nanomolar vanillin, an e-cigarette flavorant, appears to disrupt pluripotency and promote endodermal differentiation in human embryonic stem cells via TRPV4 activation
Source: Hum Reprod. 2026 Aug 12;41(10):1688–702. doi: 10.1093/humrep/deag126 (PMC13635455; doi:10.1093/humrep/deag126)
Supplement: Supplementary file 6 [file deag126_supplementary_figure_s6.pdf]

Human Reproduction, 2026, 1–1
https://doi.org/10.1093/humrep/deag126
Supplementary Data

human reproduction
OXFORD

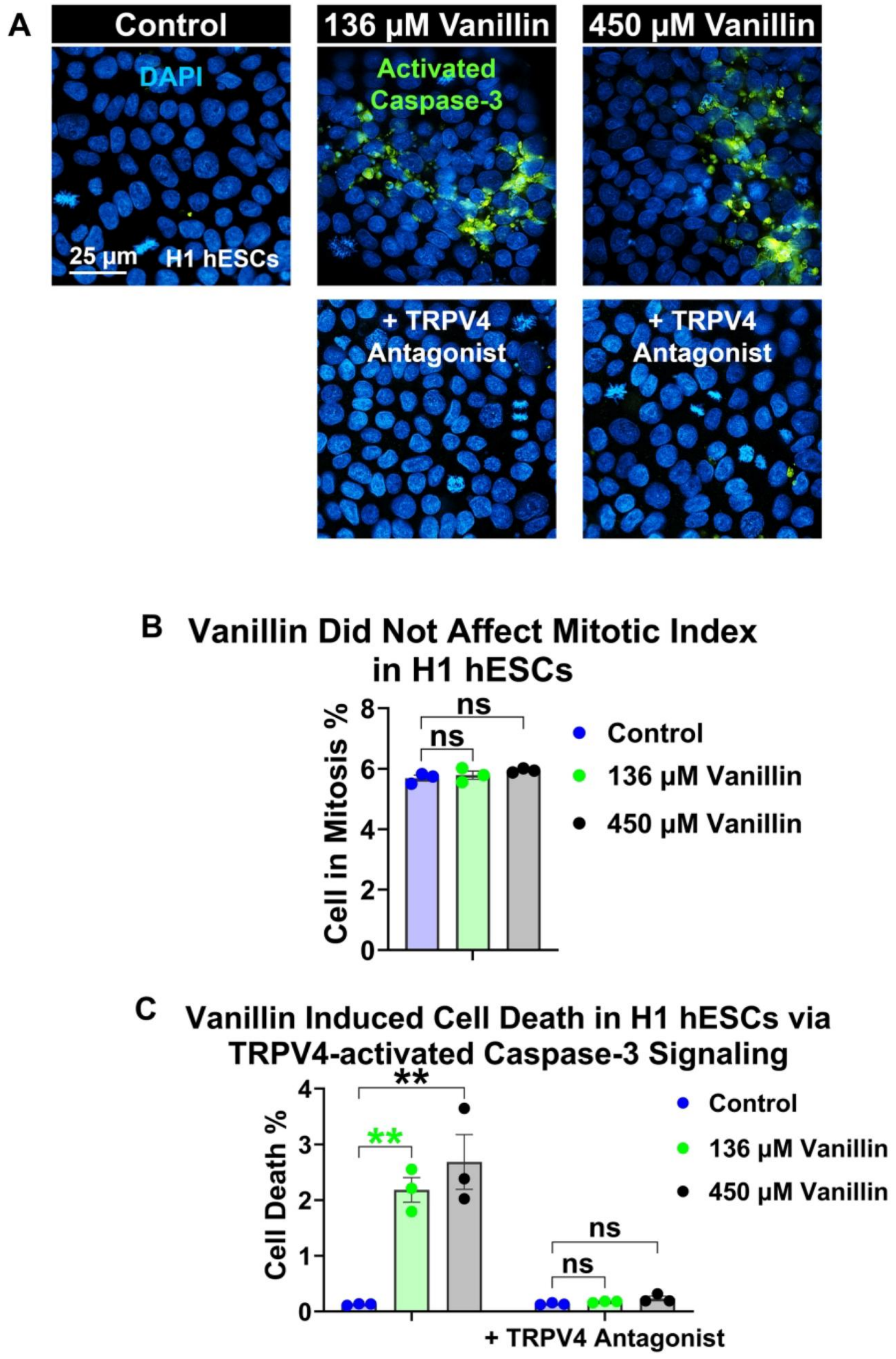

Supplementary Figure S6. Vanillin induces cell death in H1 hESCs by activation of TRPV4 channels. (**A**) Representative immunofluorescence micrographs of H1 hESCs treated with vanillin and vanillin + the TRPV4 antagonist and then labeled with an antibody against activated caspase-3 (green) to detect apoptosis. Merged micrographs include DAPI counterstaining (blue). Scale bar = 25 μm. Activated caspase-3 labeling was increased with vanillin but absent in the presence of the TRPV4 antagonist HC067047 (0.1 nM). (**B**) Quantification of the mitotic index (metaphase + anaphase) showed that vanillin did not affect mitosis in H1 hESCs. (**C**) Percentage of cell death (activated caspase-3-positive cells) following treatment with vanillin and vanillin ± the TRPV4 antagonist. Data are presented as means±SEM from three independent experiments, each with a different passage of cells. In each experiment, three colonies per group were analyzed and 100 cells were counted per colony. Statistical analysis was performed using one-way ANOVA followed by Dunnett's *post hoc* test compared to the control. ns = non-significant, $^{**}P < 0.01$.
