# Supplementary material for: Nanomolar vanillin, an e-cigarette flavorant, appears to disrupt pluripotency and promote endodermal differentiation in human embryonic stem cells via TRPV4 activation
Source: Hum Reprod. 2026 Aug 12;41(10):1688–702. doi: 10.1093/humrep/deag126 (PMC13635455; doi:10.1093/humrep/deag126)
Supplement: Supplementary file 7 [file deag126_supplementary_figure_s7.pdf]

Human Reproduction, 2026, 1–1
https://doi.org/10.1093/humrep/deag126
Supplementary Data

human reproduction
OXFORD

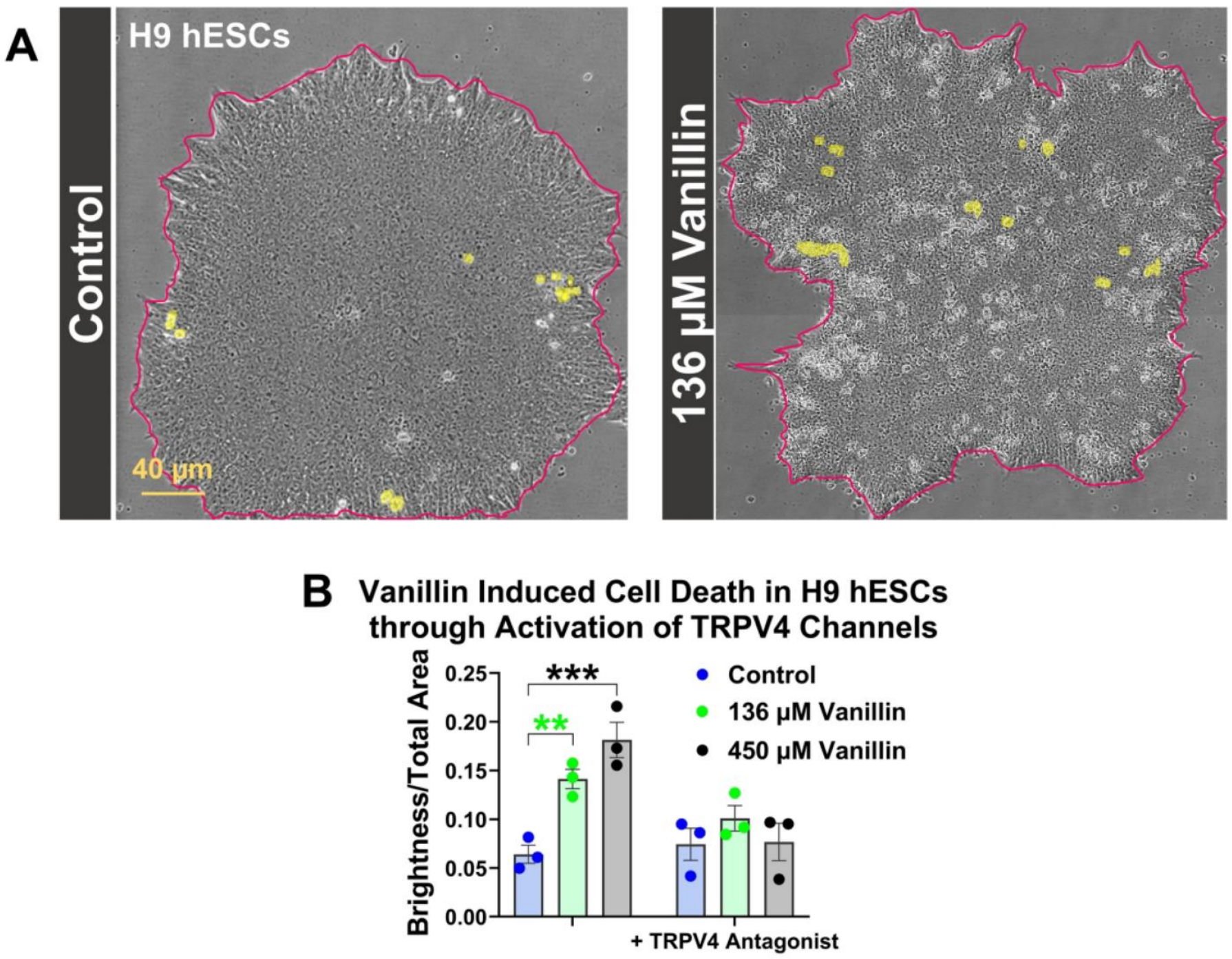

Supplementary Figure S7. **Vanillin increases h9 hESC cell death through activation of TRPV4 channels.** (**A**) Representative phase-contrast images showing dead cells (pseudocolored yellow) located on top of hESC colonies (area marked by a red outline). Cell death was quantified using the brightness-to-total area ratio feature in StemCellQC software. Scale bar = 40 µm. (**B**) Brightness-to-total area ratio was quantified over 72 h for control and vanillin-treated groups (µM range), and co-treatment with the TRPV4 antagonist HC067047 (0.1 nM) which significantly attenuated vanillin-induced hESC death. Data are presented as means±SEM from three independent experiments, each done with a different passage of cells. Statistical analysis was performed using one-way ANOVA followed by Dunnett's *post hoc* test compared to the control. $^{**}P < 0.01$, $^{***}P < 0.001$.
