# Supplementary material for: Nanomolar vanillin, an e-cigarette flavorant, appears to disrupt pluripotency and promote endodermal differentiation in human embryonic stem cells via TRPV4 activation
Source: Hum Reprod. 2026 Aug 12;41(10):1688–702. doi: 10.1093/humrep/deag126 (PMC13635455; doi:10.1093/humrep/deag126)
Supplement: Supplementary file 8 [file deag126_supplementary_materials_and_methods.pdf]

Human Reproduction, 2026, 1–2
https://doi.org/10.1093/humrep/deag126
Supplementary Data

human reproduction

OXFORD

Supplementary materials and methods

Notes on the TRPV4 antagonist and function-blocking antibody

HC067047 (Sigma Aldrich, Cat. No. SML0143) is a potent and selective antagonist of human TRPV4 channels (Dias *et al.*, 2019; Everaerts *et al.*, 2010; Thapak *et al.*, 2020; Woods *et al.*, 2021). To minimize potential off-target effects on related TRP channels, we used HC067047 at a low working concentration of 0.1 nM. Control experiments confirmed that 0.1 nM HC067047 alone had no detectable effect on intracellular Ca^{2+} levels, mitochondrial reductase activity (MTT assay), or expression of pluripotency markers.

To independently validate the pharmacological findings obtained with HC067047, we also used a function-blocking antibody directed against an extracellular epitope of TRPV4 (Alomone Labs, Cat. No. ACC-124), an approach previously established for selective blockade of TRP-channel activity (Naylor *et al.*, 2008; Miller *et al.*, 2014). The antibody was applied at 1 µM (≈150 µg/ml) for 30 min prior to vanillin exposure. This extracellular-targeting antibody inhibited vanillin-induced Ca^{2+} influx, providing a non-pharmacological confirmation of TRPV4 involvement. Under the same conditions, a rabbit isotype control IgG (Thermo Fisher Scientific, Cat. No. 02-6102) had no effect on vanillin-induced Ca^{2+} influx, confirming the specificity of the blocking effect.

Immunocytochemistry labeling and analysis

After fixation with paraformaldehyde, samples were extensively washed with DPBS, permeabilized with 0.1% Triton X-100, and blocked with 10% donkey serum to reduce non-specific binding. Cells were then incubated overnight at 4 °C with primary antibodies diluted in PBS-T (DPBS containing 0.1% Tween-20). Following several washes with PBS-T, cells were labeled with secondary antibodies Alexa fluor-488 (1:500) for 2 h at room temperature. Nuclei were counterstained using VECTASHIELD® mounting medium containing DAPI to minimize photobleaching. Fluorescence imaging was performed on a Nikon Eclipse Ti inverted microscope equipped with 20×–60× objective lenses. Images were captured using a high-resolution Andor Zyla VSC-04941 camera and processed in Nikon NIS-Elements AR and ImageJ.

Assessment of mitochondrial reductase activity via MTT assay

Following vanillin treatment, 20 µl of MTT dissolved in 5 mg/ml of DPBS were added to each well, and plates were incubated for 2 h at 37 °C. The plates were drained of the solutions, and 100 µl of dimethyl sulfoxide (DMSO) were added and gently mixed to dissolve the formazan crystals. Absorbance was measured at 570 nm using a BioTek Synergy HTX Microplate Reader.

To evaluate TRPV4 channel involvement, MTT concentration–response curves were generated in the presence of serial dilutions of the TRPV4 antagonist (HC067047). Prior to exposure to vanillin at its IC_{50} concentration, hESCs were pre-incubated with the antagonist. The IC_{50} value was determined with GraphPad Prism using nonlinear regression (dose–response-inhibition: [Inhibitor] vs response–variable slope (four parameters)). The IC_{70} was derived from the IC50 using the following equation provided by GraphPad Prism (Equation 1):

$$IC_{70} = \left(\frac{70}{100 - 70}\right)^{\frac{1}{\text{Hill Slope}}} \times IC_{50} \quad (1)$$

The Hill slope was estimated by the four-parameter nonlinear regression model. The lowest observed adverse effect level (LOAEL) was identified as the lowest concentration of vanillin that produced a significant effect compared to the control. Concentration–response curves were generated using GraphPad Prism. Three independent experiments were performed using different passages.

Time-lapse imaging and colony analysis

Following 48 h of H9 colony attachment, hESCs were exposed to vanillin at 45–450 µM concentrations for 72 h in the BioStation CT live-cell imaging incubator. Phase-contrast images were taken every 4 h, focusing on colonies that remained in the field of view for the entire incubation period. Nine colonies per condition were selected from different fields for both control and treatment groups. For antagonist experiments, cells were pre-incubated with the TRPV4 antagonist (0.1 nM HC067047) for 20 min prior to vanillin exposure. Time-lapse images were stitched into videos that captured the full 72 h using CL Quant software (Alworth *et al.*, 2010). Videos were analyzed with StemCellQC software (Zahedi *et al.*, 2016), and feature graphs were generated using GraphPad Prism. Only for detachment assay, detached H9 colonies were manually counted in the final three recorded frames.

Three colonies per condition were analyzed in each of three independent experiments (distinct passages), resulting in a total of nine colonies per group.

Assessment of cell death using live-cell imaging and brightness/total-area ratio analysis

Time-lapse images were used to quantify cell death by measuring the brightness-to-total-area ratio within individual colonies.

As H9 hESCs within a colony undergo death, they are extruded toward the colony surface, resulting in a localized increase in brightness in phase-contrast images. These bright regions correspond to dead cells (Supplementary Fig. S7). To quantify them, the bright-to-total-area feature in StemCellQC was used to calculate the number of bright pixels (above a defined intensity threshold) divided by the total colony area. An increased bright-area ratio indicates elevated cell death.

Graphs of the bright-to-total-area ratio were generated at the end of the 72-h incubation period using GraphPad Prism. Three colonies per condition were analyzed in each of three independent experiments (distinct passages), resulting in a total of nine colonies per group. This image-based metric has been previously validated as a

reliable indicator of cell death in pluripotent stem cells (Lin *et al.*, 2021; Etemadi and Talbot, 2025).

hESCs differentiation into three germ layer cells

H9 hESCs were differentiated into ectoderm, mesoderm, and endoderm lineages using the STEMdiff^{TM} Trilineage Differentiation Kit. When cultures reached 80% confluency, cells were washed with PBS and incubated with a cell dissociation reagent for 8 min at 37 °C. mTeSR^{TM} Plus medium was then added, and colonies were gently triturated to promote detachment. Detached colonies were transferred to a tube, and remaining adherent cells were rinsed with DMEM; the rinse was combined with the suspension. The cell suspension was centrifuged at 1000 rpm for 3 min, and the supernatant was discarded.

The resulting cell pellet was resuspended in single-cell plating medium supplemented with 10 μM Y-27632, and cells were gently pipetted to achieve a single-cell suspension. Cell density was optimized for each germ layer: 1×10^5 cells/ml for ectoderm and endoderm, and 2.5×10^4 cells/ml for mesoderm. Cell concentrations were determined using a hemocytometer. Cells were seeded at 250 μl per chamber in 8-well chamber slides and incubated at 37 °C for 24 h (Day 0).

On Day 1, the medium was replaced with 500 μl/well of lineage-specific STEMdiff^{TM} Trilineage Differentiation Medium. On Day 2, the medium was replaced with 250 μl/well of fresh trilineage medium. From Day 3 onward, cells were first washed with 125 μl/well of PBS (+), and the Day 2 medium change protocol was repeated daily until Day 5 for mesoderm and endoderm differentiation, and until Day 7 for ectoderm.
