# Supplementary material for: Nanomolar vanillin, an e-cigarette flavorant, appears to disrupt pluripotency and promote endodermal differentiation in human embryonic stem cells via TRPV4 activation
Source: Hum Reprod. 2026 Aug 12;41(10):1688–702. doi: 10.1093/humrep/deag126 (PMC13635455; doi:10.1093/humrep/deag126)
Supplement: Supplementary file 9 [file deag126_supplementary_table_s1.pdf]

Human Reproduction, 2026, 1–1
https://doi.org/10.1093/humrep/deag126
Supplementary Data

human reproduction

OXFORD

Supplementary Table S1. Key resources table.

Reagent resource	Source	Identifier
**Antibodies**		
TRPV4 Antibody (1:200)	Abcam, Cambridge, MA, USA	Cat. No. ab191580
TRPV1 Antibody (1:200)	Abcam, Cambridge, MA, USA	Cat. No. ab3487
TRPV4 (Extracellular) Antibody	Alomone Labs, Jerusalem, Israel	Cat. No. ACC-124
Rabbit IgG isotype Antibody	Thermo Fisher Scientific, Chino, CA, USA	Cat. No. 02-6102
Activated Caspase-3 (Asp175) (1:200)	Cell Signaling Technology, Danvers, MA, USA	Cat. No. 9661
EpCAM (CD326) (1:250)	Thermo Fisher Scientific, Chino, CA, USA	Cat. No. 14-9326-82
PAX6 Polyclonal Antibody (1:60)	Thermo Fisher Scientific, Chino, CA, USA	Cat. No. PA1-801
Human SOX17 Antibody (1:200)	R&D Systems, Minneapolis, MN, USA	Cat. No. AF1924-SP
NCAM Antibody (1:30)	Developmental Studies Hybridoma Bank, Iowa City, IA, USA	Cat. No. 5.1H11
OCT3/4 Antibody (C-10) (1:200)	Santa Cruz, Dallas, TX, USA	Cat. No. sc-5279
Donkey Anti-Rabbit Secondary Antibody, Alexa Fluor-488 (1:500)	Thermo Fisher Scientific, Houston, TX, USA	Cat. No. A21206
Donkey Anti-Mouse Secondary Antibody, Alexa Fluor-488 (1:500)	Thermo Fisher Scientific, Houston, TX, USA	Cat. No. A21202
Donkey Anti-Goat Secondary Antibody, Alexa fluor-594	Invitrogen, Carlsbad, CA, USA	Cat. No. A-11058
**Experimental models: cell lines**		
WiCell (H9; Female)/ (H1; Male)	Madison, WI, USA	Cat. No. WA09/WA01
**Culturing reagents and chemicals**		
Dulbecco's Phosphate-Buffered Saline Without Calcium or Magnesium	DPBS-; Lonza, Walkersville, MD, USA	Cat. No. 14190144
Donkey Serum	Sigma-Aldrich, St. Louis, MO, USA	Cat. No. D9663
Vectashield with DAPI	Vectashield, San Francisco, CA, USA	Cat. No. H-1200
Matrigel	Corning, New York, NY, USA	Cat. No. 354234
mTeSR Plus Medium	STEMCELL Technologies, Vancouver, BC, Canada	Cat. No. 100-0276
ReLeSR™	STEMCELL Technologies, Vancouver, BC, Canada	Cat. No. 05872
Accutase	Innovative Cell Technologies, San Diego, CA, USA	Cat. No. AT-104
Vanillin	Thermo Fisher Scientific, Chino, CA, USA	Cat. No. A11169.22
Vanillic Acid	Thermo Fisher Scientific, Chino, CA, USA	Cat. No. A12074.22
TRPV4 Antagonist (HC067047)	Sigma-Aldrich, St. Louis, MO, USA	Cat. No. SML0143
HHBS (Hanks' Buffer with 20 mM HEPES $C_8H_{18}N_2O_4S$)	AAT Bioquest, Sunnyvale, CA, USA	Cat. No. 20011
DMSO (Dimethyl Sulfoxide)	ATCC, Manassas, VA, USA	Cat. No.4-X
MTT (Methyl Thiazoyl Tetrazolium)	Sigma-Aldrich, St. Louis, MO, USA	Cat. No. M5655
Gentle Cell Dissociation Reagent	STEMCELL Technologies, Vancouver, BC, Canada	Cat. No. 100-0485
DMEM	Thermo Fisher Scientific, Chino, CA, USA	Cat. No. 11965092
Rock Inhibitor Solution (Y-27632)	STEMCELL Technologies, Vancouver, BC, Canada	Cat. No. 72304
H_2O_2	Rite Aid, Camp Hill, PA, USA	Cat. No. NDC 11822-3155-3
**Equipment**		
Nikon Eclipse Ti Inverted Microscope	Nikon Instrument, Melville, NY, USA	
Andor Zyla VSC-04941 Camera	Andor, Belfast, UK	
BioMate 3S Spectrophotometer	Thermo Fisher Scientific, Chino, CA, USA	
Nikon BioStation CT	Nikon Instruments, Melville, NY, USA	
Synergy HTX Microplate Reader	BioTek, Winooski, VT, USA	
**Consumables**		
8-Well Chamber Slides	Ibidi, Gräfelfing, Germany	Cat. No. 80826-90
96-Well Clear Bottom Black Plates	Greiner Bio-One, Monroe, NC, USA	Cat. No. 655090
**Software and algorithms**		
Nikon NIS-Elements AR	Nikon	v.4.60.00
MATLAB R2024b	MATLAB	v.24.2
MATLAB Runtime R2024b	MATLAB	v.24.2
CL Quant	Nikon	v.3.10
StemCellQC	Talbot Lab	v.2-17 https://talbotlab.ucr.edu/stemcellqctm
Prism 10	Graphpad	v.10.4.2
ImageJ	NIH	v.1.54f
**Critical commercial assays**		
Fluo-8 No Wash Calcium Assay Kit	Abcam, Cambridge, MA, USA	Cat. No. ab112129
STEMdiff™ Trilineage Differentiation Kit	STEMCELL Technologies, Vancouver, BC, Canada	Cat. No. 05230
